# Telomere-to-Telomere Assembly of the *Cordyceps militaris* CH1 Genome and Integrated Transcriptomic and Metabolomic Analyses Provide New Insights into Cordycepin Biosynthesis Under Light Stress

**DOI:** 10.3390/jof11060461

**Published:** 2025-06-18

**Authors:** Yang Yang, Jingjing Huang, Gangqiang Dong, Xuebo Hu

**Affiliations:** 1 Institute for Medicinal Plants, College of Plant Science and Technology, Huazhong Agricultural University, Wuhan 430070, China; life333@webmail.hzau.edu.cn; 2 Innovation Academy of International Traditional Chinese Medicinal Materials, Huazhong Agricultural University, Wuhan 430070, China; 3 Amway (China) Botanical R&D Center, Wuxi 214115, China; evelyn.huang@amway.com (J.H.); tony.dong@amway.com (G.D.)

**Keywords:** *Cordyceps militaris* CH1, telomere-to-telomere, phylogenetic evolution, comparative genomics, transcriptome and metabolome, cordycepin biosynthesis

## Abstract

*Cordyceps militaris*, a model species in the genus Cordyceps, is widely distributed globally and is known for its significant medicinal value. It has been traditionally used in Chinese medicine to enhance immunity, alleviate fatigue, and treat tumors, among other therapeutic purposes. Here, we successfully assembled a telomere-to-telomere (T2T) level genome of *C. militaris* CH1 using PacBio HiFi and Hi-C technologies. The assembled genome is 32.67 Mb in size, with an N50 of 4.70 Mb. Gene prediction revealed a total of 10,749 predicted genes in the *C. militaris* CH1 genome, with a gene completeness of 99.20%. Phylogenetic analysis showed the evolutionary relationship between *C. militaris* CH1 and other *Cordyceps* species, suggesting that the divergence between this strain and *C. militaris* ATCC 34164 occurred approximately 1.36 Mya. Combined transcriptomic and metabolomic analyses identified 842 differentially expressed genes and 2052 metabolites that were significantly altered under light stress, primarily involving key pathways related to amino acid metabolism, purine metabolism, and secondary metabolite biosynthesis. Joint analysis of genes and metabolites revealed 79 genes coding for enzymes associated with the synthesis of adenine and adenosine, with the expression of 52 genes being upregulated, consistent with the accumulation trends of adenine and adenosine. Four gene clusters related to the synthesis of cordycepin were identified, with a significant upregulation of cns3 (*FUN_003263*), suggesting that light stress may promote cordycepin biosynthesis. This comprehensive analysis not only provides new insights into the genomics, metabolomics, and functional gene research of *C. militaris* CH1 but also offers a potential biological foundation for understanding the synthesis mechanisms of cordycepin and its efficient production.

## 1. Introduction

The genus *Cordyceps* belongs to the Ascomycota phylum and consists of parasitic fungi, with all known species being endoparasitic, primarily feeding on insects or arthropods, while a few species parasitize other fungi [1]. *Cordyceps militaris* is the type species of the genus *Cordyceps* and is widely distributed across the globe [2]. In recent years, bioactive compounds from *C. militaris*, especially cordycepin and polysaccharides, have become a research hotspot. Studies have shown that *C. militaris* possesses significant medicinal value, and it has been used in traditional Chinese medicine for immune enhancement, anti-fatigue, anti-tumor, and other therapeutic effects [3,4,5]. In the 1960s, Basith Mumtaz successfully cultivated *C. militaris* under artificial conditions and began producing the fruiting bodies of the fungus, which was later developed into large-scale production [6]. Currently, *C. militaris* has become an alternative to the increasingly scarce wild *C. sinensis*, with promising applications in various fields. With the development of biotechnology, the artificial cultivation technology for *C. militaris* has continuously advanced, offering broad prospects in the pharmaceutical and health supplement industries [7]. However, in-depth research on the biological mechanisms and active ingredients of *C. militaris* remains a critical issue that needs to be addressed. Furthermore, cultivated strains available in the market currently have limited genetic diversity, often leading to discrepancies in products with the same name, and most of these strains exhibit low levels of active compounds, reducing their market competitiveness.

*C. militaris* contains a variety of bioactive compounds, including polysaccharides, cordycepin(3′-deoxyadenosine), cordycepic acid (mannitol), ergosterol, adenosine, and N6-(2-hydroxyethyl) adenosine, among other nucleosides [8]. Among these, cordycepin is considered the main active component of *C. militaris* [9]. The biosynthesis of cordycepin occurs through two main pathways [10]. The first pathway involves the biosynthesis of nucleosides/nucleotides. Glucose is converted to adenosine monophosphate (AMP) through glycolysis, the pentose phosphate pathway, and purine biosynthesis, which is subsequently converted to adenosine diphosphate (ADP) by adenosine kinase (ADEK). AMP is then reduced to 3′-deoxyadenosine-5′-diphosphate (3′-dADP) by ribonucleotide reductase (RNR), followed by conversion to 3′-deoxyadenosine-5′-monophosphate (3′-dAMP) and finally dephosphorylated to cordycepin by 5′-nucleotidase (NT5E) [11]. The second pathway involves the regulation of cordycepin biosynthesis by the gene cluster cns1-cns4 [12]. Four genes (*Cns1–Cns4*) involved in cordycepin biosynthesis were identified through whole-genome sequencing of *C. militaris*. These genes encode proteins that promote cordycepin metabolism through various conserved domains. Specifically, these genes encode key enzymes, including oxidoreductases/dehydrogenases (cns1), metal-dependent phosphohydrolases (cns2), nucleoside/nucleotide kinases (cns3), and ABC multidrug transporters (cns4). Adenosine-3′-monophosphate (3′-AMP) is the direct precursor of cordycepin and is converted into 3′-AMP by the nucleoside/nucleotide kinase domain of Cns3, or through phosphorylation of 2′,3′-cyclic nucleotide (2′,3′-cAMP). Cns2 then catalyzes the conversion of 3′-AMP to the intermediate 2′-carbonyl-3′-deoxyadenosine (2′-C-3′-dA), which is further catalyzed by Cns1 to form cordycepin. It is important to note that the cooperation between Cns1 and Cns2 is crucial for cordycepin biosynthesis. Pentostatin (PTN), an adenosine deaminase inhibitor, regulates the content of cordycepin in *C. militaris* by preventing its conversion into 3′-deoxyinosine (3′-dI) [12]. Furthermore, light plays a critical role in the synthesis of cordycepin. Studies have shown that under light conditions, carotenoids respond by upregulating genes related to the AMP pathway, increasing the expression of 2-hydroxyglutarate-related genes, and promoting the biosynthesis of cordycepin [13]. Despite numerous hypotheses about the biosynthesis of cordycepin, a comprehensive understanding of these pathways remains elusive.

The genome sequencing of *C. militaris* has provided important data for understanding its biological characteristics, pharmacological effects, and the mechanisms underlying the production of its bioactive compounds. In recent years, with the rapid development of high-throughput sequencing technologies, several research teams have sequenced and analyzed the genome of *C. militaris*, laying the foundation for further understanding its genetic features. The first genome sequencing of *C. militaris* used shotgun sequencing and achieved a coverage of 147×, resulting in the assembly of 33 scaffolds with an N50 of 4.6 Mb and a total genome size of 32.2 Mb [14]. With the application of PacBio single-molecule real-time (SMRT) sequencing technology, the complete genome of *C. militaris* was sequenced with a coverage exceeding 300×, resulting in the assembly of 14 contigs, with a chromosomal-level genome size of 32.57 Mb and an N50 of 2.86 Mb [15]. The genome of the *C. militaris ATCC* strain further improved assembly quality, with only seven contigs and a total genome size of 33.6 Mb [16]. Currently, T2T (telomere-to-telomere) genome assembly has become an important direction in genomic research. It aids in identifying unique genes and structural variations in the “dark matter” regions of the genome, such as centromeres, transposable elements (TEs), and segmental repeat sequences. The publication of the first complete, gap-free human genome sequence paved the way for understanding human health and the unique characteristics of our species [17]. Gapless genomes have also been completed in plants, such as Arabidopsis [18], sorghum [19], and rice [20], and the T2T genome approach has been applied to fungi, such as *Ganoderma leucocontextum* [21] and *Ustilaginoidea virens* [22]. Therefore, filling the gaps in the *C. militaris* genome and constructing a high-quality T2T genome will contribute to the comprehensive understanding of its genetic characteristics and related biological research.

Here, we integrated PacBio and Hi-C sequencing technologies to successfully construct a high-quality T2T-level genome of *C. militaris CH1*. Structural analysis of the genome allowed us to clarify its phylogenetic position within the fungal kingdom, and comparative genomics revealed the unique characteristics of the Chinese *C. militaris CH1*. Furthermore, through transcriptomics and metabolomics analyses, we explored how light regulates the metabolism of cordycepin and the expression of related genes. This comprehensive genomic analysis provides valuable molecular data for future systematic studies on the molecular mechanisms of *C. militaris CH1*.

## 2. Materials and Methods

### 2.1. Sampling Information

*Cordyceps militaris* mycelium was inoculated into potato dextrose broth (PDA) in conical flasks and then cultured at 26 °C with shaking at 150 rpm for 5 days. The cultures were subsequently rapidly frozen in liquid nitrogen. Genomic DNA was extracted using the CTAB method [23], and after assessing the quality through 1% agarose gel electrophoresis, the concentration of DNA was accurately measured using a Qubit 3.0 fluorometer. Once the DNA concentration met the required standards, this high-quality genomic DNA was used for genome sequencing and chromosome-level genome assembly.

### 2.2. Genome Sequencing and Assembly

To achieve high-quality genome assembly of *Cordyceps militaris* CH1, a combination of PacBio HiFi and Illumina sequencing technologies was employed. For Illumina sequencing, paired-end libraries were constructed on the Illumina HiSeq X platform. For long-read sequencing, SMRTbell libraries with an average insert size of 20 kb were prepared and sequenced using the PacBio RSII platform to generate high-fidelity reads. In parallel, a Hi-C library was also constructed and sequenced on the Illumina HiSeq X platform to facilitate chromosome-level scaffolding. For the raw sequencing data, sequences with Q30 values below 85% were filtered out to ensure data quality.

Genome assembly was first performed using Hifiasm (v0.19.9) [24] with default parameters to assemble the PacBio HiFi reads into contigs. Base-level corrections were subsequently applied using NextPolish (v1.4.1) [25], which incorporated Illumina short reads in multiple polishing rounds. Hi-C data were processed with HapHiC (v1.0.3) [26] for scaffolding and alignment, and manual refinement of chromosome anchoring was performed using Juicebox (v1.13) [27]. The completeness and integrity of the final chromosome-level genome assembly were evaluated with BUSCO (v5.1.2) [28], using the fungal lineage dataset fungi_odb10.

### 2.3. Genome Size Estimation

The genome size of *C. militaris* CH1 was assessed using the K-mer counting approach [29]. The Jellyfish tool (v2.3.1) was employed to calculate K-mer frequency distributions from cleaned sequencing data, selecting an optimal K-mer size. Genome size, heterozygosity, and repeat content were evaluated using GenomeScope 2.0 [30], combining peak K-mer frequencies with GenomeScope analysis.

### 2.4. Gene Prediction and Annotation

In the identification of repetitive sequences in the genome of *Cordyceps militaris* CH1, this study adopted an integrative strategy that combined de novo predictions with reference database analyses. RepeatModeler (v2.0.5) [31] was utilized to conduct de novo prediction of repetitive elements in the genome, and the results were masked using RepeatMasker (v4.0.9) [32]. To enhance the coverage of known repetitive elements, the RepBase database was specifically employed to systematically supplement any potentially overlooked conserved repetitive elements from the de novo prediction. The simple repeat sequences were collected and counted.

Gene prediction was conducted using an integrative strategy that combined de novo prediction, homology-based alignment, and transcriptome-guided methods. First, based on the genomic characteristics of the fungi. The Augustus (v3.4) [33] was used for optimized modeling, supplemented by the machine learning framework SNAP (v2.0) [34] as an auxiliary prediction tool. Fine annotation of homologous genes was achieved through alignment with the genomes of closely related fungi using GeMoMa (v1.9) [35]. The results from various prediction methods were integrated using EVidenceModeler (v1.1) [36] to construct a non-redundant gene set. Functional annotation was completed through multi-dimensional comparisons: sequence similarity searches were performed in the NCBI NR, UniProt, and InterPro databases; molecular functions, biological processes, and cellular components were annotated based on the Gene Ontology (GO) framework [37]; and potential biochemical functions of the genes were analyzed through KEGG [38] metabolic pathways. For the recognition of non-coding RNAs, Infernal (v1.1.2) [39] was used to predict snRNA in comparison with the Rfam database, tRNAscan (v2.0.9) [40] was employed to detect tRNA. BLASTn (v2.7.1) [41] was utilized for precise localization of rRNA sequences.

### 2.5. Gene Family Clustering and Phylogenetic Analysis

Based on the phylogenetic relationship of the *C. militaris* species, genomic information for 11 fungal strains was selected from the China National GeneBank (CNGB), including *C. militaris* ATCC, *Amanita muscaria*, *Beauveria bassiana*, *Cordyceps cateniannulata*, *Cordyceps cicadae*, *Cordyceps fumosorosea*, *Cordyceps javanica*, *Cordyceps pruinosa*, *Cordyceps tenuipes*, *Curvularia cyperi*, and *Lecanicillium fungicola*. OrthoFinder (v2.5.4) [42] was employed to perform orthologous gene clustering analysis on the proteins of 12 fungi. Based on the identified single-copy gene clusters, multiple sequence alignment was carried out using MAFFT (v7.471) [43], and a phylogenetic tree was constructed using the maximum likelihood method in IQ-TREE (v2.0.6) [44] with 1000 bootstrap replicates to support the branch nodes. The species divergence time was jointly estimated by integrating data from the TimeTree database and the mcmctree module of the PAML package [45]. The expansion and contraction events of gene families were quantitatively analyzed using the evolutionary model provided by CAFE (v4.2.1) [46], and enrichment analyses for expanded and contracted families were conducted with using the R package clusterProfiler (v4.14.0) [47] with default parameters.

### 2.6. Comparative Genomic Analysis

The genome was aligned to the reference genome using MUMmer4 [48], and the positions of SNPs and INDELs were identified. Subsequently, their numbers were counted for each chromosome. Data processing and plotting were performed using R (v4.3.2).

Using MCScanX to perform genomic collinearity analysis (v1.0) [49], with BLASTp (v2.7.1) (e < 1 × 10^−5^) [41] used for collinearity analysis.

### 2.7. Identification of CAZymes, CYP450s, and Secondary Metabolite Clusters

The P450 domain model (Pfam ID: PF00067) was downloaded from the Pfam database (http://pfam.xfam.org/, accessed on 12 November 2024). Subsequently, HMMER 3.0 [50] was used to search all *C. militaris* CH1 protein sequences based on this P450 reference domain model with a threshold of (E < 1 × 10^−5^). The initially screened CYP450 genes were then submitted to the SMART database (https://smart.embl.de/, accessed on 13 November 2024) for manual identification. Finally, the identified P450 genes were confirmed as the cytochrome P450 genes of *C. militaris* CH1. The protein of *C. militaris* CH1 was submitted to the website (https://bcb.unl.edu/dbCAN2/ accessed on 18 November 2024) for prediction. HMMER was used to search the candidate CAZymes proteins based on the characteristic domains of key CAZymes, which were selected with E-values ≤ 1 × 10^−5^. Proteins matching specific reference CAZymes domains were then considered as corresponding CAZymes genes. Secondary metabolite clusters were predicted using the website (https://fungismash.secondarymetabolites.org, accessed on 12 November 2024).

### 2.8. Transcriptome Analysis

During the active growth phase, *C. militaris* CH1 mycelia were incubated for 5 days under dark and light (using the light mode provided by the instrument) conditions in a ZQWY-200 incubator (Zhichu Instruments, Shanghai, China). Afterward, the mycelium was rapidly frozen in liquid nitrogen, and RNA was extracted using the TRI-zol method [51] and sequenced on the Illumina HiSeq 4000 sequencing platforms. The raw sequencing data were first subjected to quality control using FastQC, and then low-quality bases were removed using fastp (v0.23.4) with default parameters [52]. High-quality sequences were aligned to the *C. militaris* CH1 genome using HISAT2 (v2.2.1) [53]. Transcript assembly and gene expression quantification were carried out with StringTie (v2.2.1) [54], followed by FPKM calculation using Cufflinks (v2.2.1) [55]. Differential expression analysis was performed using the limma package in R. Genes showing a log_2_ (fold change) ≥ 2 or ≤−0.5 with a *p*-value < 0.05 were considered significantly upregulated or downregulated, respectively. The KEGG and GO enrichment analysis of the target genes was performed using ClusterProfiler (v4.14.0), with pathways having a *p*-value < 0.05 considered significantly enriched.

### 2.9. Metabolome Analysis

*C. militaris* CH1 mycelium was treated under dark and light stress conditions for 5 days, with 6 biological replicates per treatment group. Metabolite extraction involved grinding 50 mg of frozen mycelium in ice-cold methanol/water (7:3, *v*/*v*), followed by sonication using a Branson B2510 ultrasonic cleaner (Branson Ultrasonics, Wilmington, NC, USA) for 10 min at 4 °C. The samples were then incubated at −20 °C for 1 h to precipitate proteins. After incubation, the supernatant was centrifuged at 13,000 rpm for 15 min at 4 °C. The supernatant was dried in a vacuum concentrator at 4 °C and re-suspended in 100 μL of acetonitrile/water (1:1, *v*/*v*) for analysis using liquid chromatography-mass spectrometry (LC-MS) [56]. The analysis was performed on an Agilent 1290 LC system and a Thermo Fisher Q Exactive Orbitrap mass spectrometer (Thermo Fisher Scientific, Wilmington, NC, USA) in both positive and negative ion modes [57]. Data were processed for peak identification, denoising, normalization, and statistical analysis, with PCA, PLS-DA, and the Wilcoxon test applied. Differentially expressed genes were subjected to KEGG pathway enrichment analysis using the clusterProfiler, with enriched pathways visualized through dotplot to highlight their significance and gene ratios.

### 2.10. qRT-PCR Analysis Method

To validate the reliability of the transcriptome data, four genes related to cordycepin synthesis (*FUN_003262*, *FUN_003263*, *FUN_003264*, *FUN_003266*) and four adenine synthesis genes (*FUN_000548*, *FUN_000593*, *FUN_005768*, *FUN_009691*) were selected for qRT-PCR analysis. These genes were identified as significantly differentially expressed between light and dark stress conditions based on the transcriptome analysis.

High-quality RNA was extracted from *C. militaris* CH1 mycelium subjected to light and dark treatments using the Fungal Total RNA Isolation Kit (Sangon Biotech Co., Ltd., Shanghai, China). Reverse transcription was performed using SuperScript™ IV reverse transcriptase (Thermo Fisher Scientific, Wilmington, NC, USA), with Oligo(dT) primers selected to anneal to the poly(A) tail of mRNA. In the reaction, 1 µg of RNA was mixed with the primers and reaction buffer and incubated at 50 °C for 60 min to synthesize cDNA. Actin was used as the reference gene, and specific primers (Table S1) for the relevant genes were designed using Primer Premier 7.0 [58]. The fold change analysis of the mycelium from different treatments was calculated using the 2^−ΔΔCt^ method [59]. An unpaired *t*-test was conducted using R (v4.3.2) to calculate the *p*-values, and plotting was also performed using R (v4.3.2).

## 3. Results

### 3.1. Genome Sequencing and Assembly Analysis

**Figure 1:**
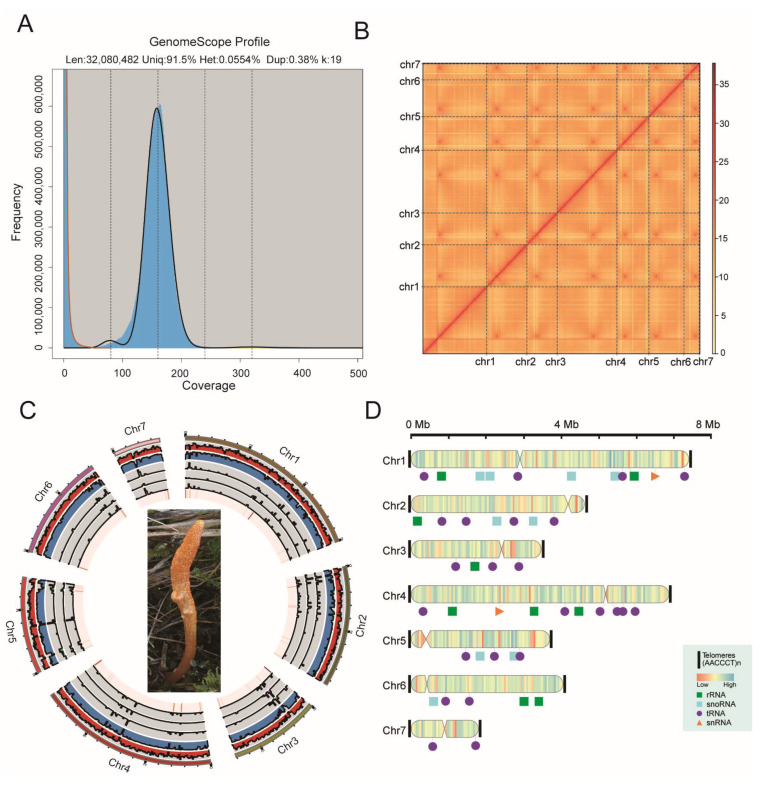
The telomere-to-telomere (T2T) genome assembly of *C. militaris* CH1. (**A**) The genomic survey map of *C. militaris* CH1 shows the distribution of different k-mers in the density plot. The black line represents the overall model fitting curve, while the red line indicates the erroneous K-mer distribution curve. (**B**) The Hi-C interaction heatmap for *C. militaris* CH1 indicates that the stronger the interaction, the redder the color, and conversely, the weaker the interaction, the more subdued the color. (**C**) The circos plot representing the genomic features of *C. militaris* CH1, with the outermost layer representing the chromosomes and the colored areas indicating coding regions. A 1 kb sliding window was used for segmentation, and the heatmap and bar chart from outer to inner layers represent GC content, gene density, density of tandem repeats, density of transposable elements, density of LTR_Gypsy, and density of LTR_Copia, respectively. The innermost lines illustrate the synteny of *C. militaris* CH1 itself. (**D**) The T2T assembly results for *C. militaris* CH1, including the positions of the telomeres and non-coding RNAs.

To obtain high-quality genome data of *Cordyceps militaris* CH1, we performed deep sequencing of the *C. militaris* CH1 genome using a combination of PacBio HiFi and Hi-C sequencing technologies. PacBio HiFi sequencing was employed to generate approximately 5.77 Gb of sequencing data, providing a coverage depth of 178.03× the genome size (Table S2). The average read length was 17.74 kb, with the longest read reaching 49.02 kb. To assemble the genome at the chromosomal level, Hi-C technology was used to generate three-dimensional structural data of the genome. After filtering the raw Hi-C data and removing adapter sequences, approximately 32.47 Gb of high-quality data were obtained, with a coverage depth of 100.19× and a quality score (Q30) of 94.37%, indicating high sequencing quality (Table S3). Based on the PacBio HiFi sequencing data, the genome size of *C. militaris* CH1 was estimated. K-mer frequency distribution analysis revealed an estimated genome size of 30.98 Mb, with 1.29% of the genome consisting of repetitive sequences (Figure 1A).

High-quality PacBio HiFi data were used for the genome assembly. The final assembled genome size was 32.67 Mb, with 10 contigs, an N50 length of 4.70 Mb, and a GC ratio of 57.68%, which closely matched the K-mer estimated value (Table S4). To further achieve a high-quality chromosome-level genome of *C. militaris* CH1, Hi-C sequencing data were incorporated, successfully anchoring the 10 contigs onto seven chromosomes. The Hi-C interaction heatmap showed strong internal interactions within each chromosome, confirming the accuracy of the assembly (Figure 1B). Telomere repeat sequence (AACCCT) was identified on both ends of seven chromosomes, and a T2T-level of the *C. militaris* CH1 genome was successfully assembled (Figure 1D). The chromosome-level genome of *C. militaris* CH1 obtained had a size of 32.41 Mb, with an anchoring rate of 99.2% (Table S5). The genome circos plot displayed the size information of the seven high-quality chromosomes. Among these chromosomes, all were assembled without gaps from telomere to telomere, Furthermore, the genome coverage map demonstrates that all chromosomes are uniformly covered, which further confirms the completeness and accuracy of the genome assembly (Figure S1). BUSCO (Benchmarking Universal Single-Copy Orthologs) analysis was used to assess the genome’s completeness, showing a completeness score of 98.10%, Through comparisons with other species of *C. militaris*, it is evident that *C. militaris* CH1 exhibits the highest level of completeness score, indicating that the genome assembly has highest quality and accuracy (Table S6, Figure S2).

### 3.2. Genome Annotation

Based on the genome assembly data of *C. militaris* CH1, this study conducted gene structure annotation through the integration of self-prediction and homology-based alignment strategies. Systematic analysis revealed a total of 10,749 protein-coding genes, with an average sequence length of 1976.97 bp. Functional element annotation indicated the presence of 29,460 coding sequences (CDS) within the genome, with an average length of 561.96 bp. During the gene structure analysis, 30,942 exonic elements were identified, exhibiting an average base length of 714.78 bp, which reflects the typical gene structure features of eukaryotes (Table 1). Through in-depth annotation, a total of 19,367 intronic elements were identified, with an average length of only 79.44 bp, consistent with the evolutionary characteristic of short introns in fungal genomes. To validate the reliability of gene predictions, an assessment based on the BUSCO framework showed a completeness of 99.20% for single-copy orthologs, indicating a high quality of genome annotation. At the level of non-coding RNA annotation, 185 tRNA genes were systematically detected, with a total length of 10,925 bp and an average length of 59.05 bp. Additionally, 34 rRNA gene clusters were identified, comprising 24 copies of 8S rRNA, 5 copies of 18S rRNA, and 5 copies of 28S rRNA, with a total length of 42,017 bp and an average length of 1235.79 bp per gene, demonstrating a typical multi-copy distribution pattern of ribosomal RNA (Table S7).

**Figure 2:**
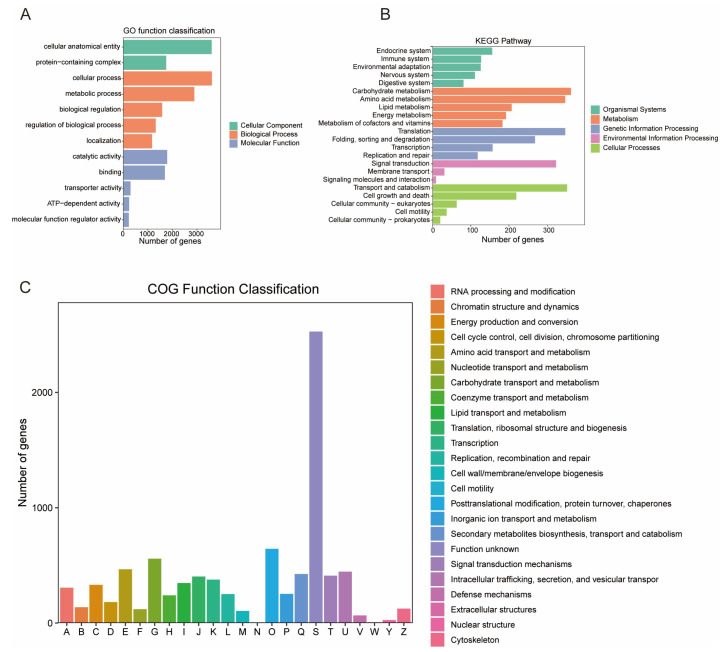
A bar chart for gene annotation of *C. militaris* CH1. (**A**) GO annotation statistics chart displays 12 significantly enriched terms categorized by biological process (BP), cellular component (CC), and molecular function (MF) using color coding. (**B**) KEGG pathway annotation chart lists the names of metabolic pathways on the vertical axis, while the horizontal axis quantifies the number of annotated genes in each pathway. (**C**) COG functional classification shows the functional categories of genes on the horizontal axis, with the vertical axis representing the number of genes in each functional category.

Functional annotation of protein-coding genes in the *C. militaris* CH1 genome was performed based on the NR, Swiss-Prot, KEGG, and GO databases. The results indicated that 98.67% of the genes were successfully annotated (Table S8). Through systematic analysis using the GO, KEGG, and COG databases, hierarchical classification of gene functions and reconstruction of metabolic networks were achieved. The GO annotation system analyzed 10,338 functional entries across 3851 genes, covering three main categories: molecular function, cellular component, and biological process (Figure 2A). In terms of cellular components, there was a significant enrichment in anatomical entity protein complexes; biological processes were predominantly represented; and molecular functions were primarily concentrated on specific activities. The KEGG pathway annotation further analyzed 292 metabolic pathways involving 2696 genes, with core pathways related to carbohydrate metabolism, amino acid metabolism, translation processes, signal transduction, and transport and catabolism (Figure 2B). Notably, the COG classification system systematically categorized 8256 genes, identifying 128 functional clusters, of which 385 genes were closely associated with the synthesis, transport, and catabolism of secondary metabolites (Figure 2C).

In the *C. militaris* CH1 genome, repetitive sequences account for 5.99% (Table S9), with transposable elements being the dominant component. Retrotransposons make up the core component at 4.51%, with long terminal repeat sequences (LTRs) comprising 4.50%. Notably, the Ty1/Copia family (0.87%) and the Gypsy/DIRS1 family (0.02%) are representative of this group. DNA transposons account for 0.01%, contributing to the structural framework of transposable elements alongside LTRs. Simple sequence repeats (SSRs) are widely distributed throughout the genome, with a total proportion of 1.25%. Further analysis indicates that SSRs exhibit differential distribution characteristics in intergenic regions compared to coding regions, suggesting their potential involvement in regulating gene expression and chromatin dynamics.

### 3.3. Evolutionary Analysis of C. militaris CH1

**Figure 3:**
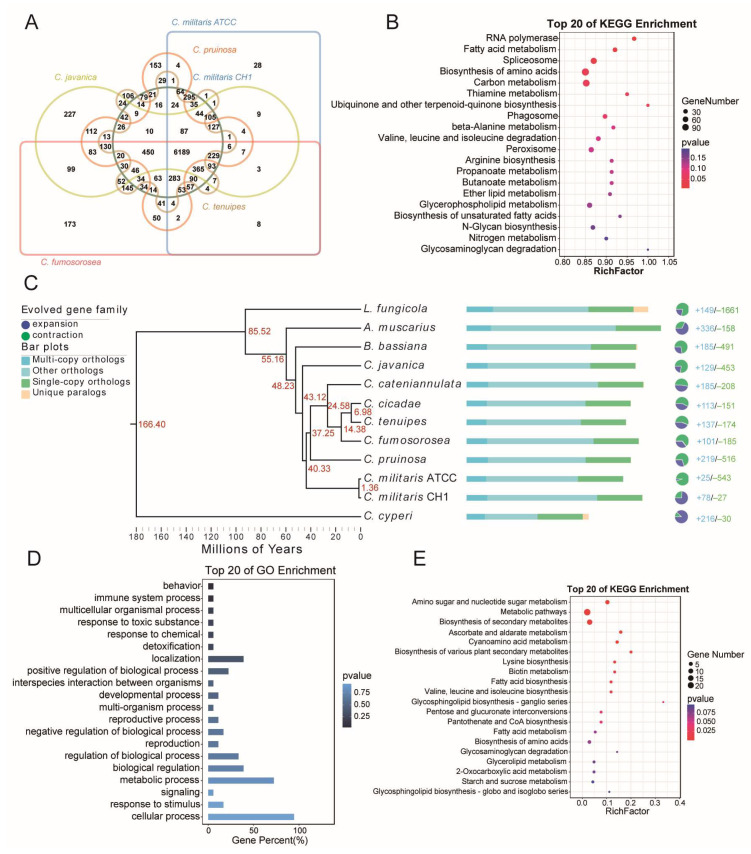
*C. militaris* CH1 gene family clustering and phylogenetic analysis with closely related species. (**A**) The Venn diagram depicts the gene families of *C. militaris* CH1 in comparison with *C. pruinosa*, *C. fumosorosea*, *C. tenuipes*, *C. militaris* ATCC, and *C. javanica*, highlighting the number of unique and shared gene families. (**B**) KEGG pathway enrichment of shared genes across six species. (**C**) The phylogenetic tree of *C. militaris* CH1 and 11 other fungi, with *C. cyperi* as the outgroup; the red numbers indicate divergence times, and the bar chart shows the number of different types of gene families in each fungus, including single-copy orthologs, multi-copy orthologs, unique paralogs, and other orthologs. The blue portion of the pie chart represents the number of expanded gene families, while the green portion indicates the number of contracted gene families. (**D**) The bar chart shows the GO functional enrichment of the expanded gene families of the *C. militaris* CH1 genome. (**E**) The dot plot illustrates the KEGG enrichment of the expanded gene families of the *C. militaris* CH1 genome.

To investigate the evolutionary relationship between *C. militaris* CH1 and other species in the genus Cordyceps, we performed gene family clustering analysis using *C. pruinosa*, *C. fumosorosea*, *C. tenuipes*, *C. militaris* CH1, *C. militaris* ATCC, and *C. javanica* (Figure 3A). The results revealed a total of 6189 shared gene families across these six species. These shared genes were significantly enriched in several KEGG pathways, including “RNA polymerase”, “spliceosome”, “fatty acid metabolism”, “biosynthesis of amino acids”, and “carbon metabolism” (Figure 3B). To further explore the evolutionary relationship between *C. militaris* CH1 and other fungi, 12 fungal species were selected for gene family clustering analysis. A total of 142,742 genes were identified, with 133,122 genes (93.3%) involved in clustering, while the remaining 9620 genes (6.7%) were not assigned to any cluster (Table S10). Additionally, 11,698 gene families were recognized, including 440 species-specific families consisting of 1498 genes, which account for 1.0% of the total gene pool. Among these clusters, 2950 genes were shared by all species, and 2064 genes were identified as single-copy gene families. To further investigate the phylogenetic relationships among 12 species, a phylogenetic tree based on the 2064 single-copy gene families was constructed (Figure 3C). The analysis showed that *C. militaris* CH1 and *C. militaris* ATCC are closely related, forming an independent branch. Another branch, consisting of *C. pruinosa*, *C. fumosorosea*, *C. tenuipes*, *C. cicadae*, and *C. cateniannulata*, was identified as a sister group to the branch of *C. militaris* CH1 and *C. militaris* ATCC. To infer the timing of species divergence, this study integrates nucleotide substitution rates from fungal phylogenetic research parameters and paleobiological calibration markers, constructing a molecular clock model for systematic evolutionary inference. The analysis results indicate that the early divergence of the genus Cordyceps can be traced back to approximately 43.12 million years ago (Mya). Notably, the intraspecific divergence event of *C. militaris* CH1 and *C. militaris* ATCC occurred around 1.36 million years ago (Figure 3C).

Phylogenetic tree-based lineage evolution analysis reveals a significant asymmetrical pattern of gene family expansion and contraction in the *Cordyceps militaris* CH1 genome, with 78 gene families experiencing expansion events and 27 families showing signs of contraction (Figure 3C). This differential evolutionary pattern suggests that the species may have undergone specific environmental adaptation and selective pressures during its evolutionary process. GO enrichment analysis revealed that the expanded gene families were significantly enriched in pathways related to “behavior”, “immune system process”, “multicellular organismal process”, “response to toxic substances”, and “response to chemicals” (Figure 3D). Additionally, KEGG enrichment analysis showed that the expanded gene families were notably associated with pathways involved in “amino sugar and nucleotide sugar metabolism”, “metabolic pathways”, and “biosynthesis of secondary metabolites” (Figure 3E).

### 3.4. Comparative Genomic Analysis of C. militaris CH1

**Figure 4:**
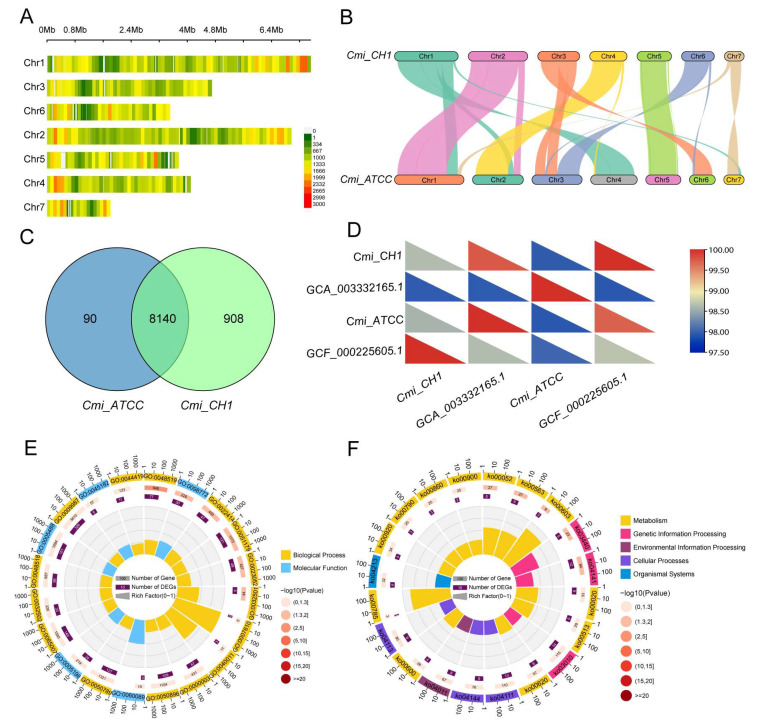
Comparative genomics analysis of intra-species and inter-species. (**A**) SNP density plot with SNP counts calculated in 0.1 Mb sliding windows. (**B**) Comparative genomic analysis based on chromosomes. (**C**) Clustering analysis of all gene families. (**D**) Genomic similarity comparison. (**E**) GO enrichment analysis of genes unique. (**F**) KEGG enrichment analysis of genes unique. All analyses were completed between *C. militaris* CH1 (Cmi_CH1) and *C. militaris* ATCC (Cmi_ATCC), except that GCA_003332165.1 and GCF_000225605.1 were included in (**D**).

To investigate the genomic characteristics of *C. militaris* CH1, a comparative genomic analysis was performed (Table S11). SNP identification was carried out on the whole genomes of *C. militaris* CH1 and the American strain of *C. militaris* ATCC (Figure 4A). The results indicate the presence of numerous differences between the two strains. Specifically, a total of 366,223 SNPs were identified, of which 142,532 are located in exon regions. After excluding 48,911 synonymous mutations, a total of 7836 genes are involved. In addition, 83,616 INDELs were identified, with the majority located in the upstream regions (Figure S3). Respectively, further comparative genomic analysis based on whole-genome protein sequence alignment revealed extensive synteny between *C. militaris* CH1 and *C. militaris* ATCC, with a synteny ratio as high as 89.2%. Some chromosomes exhibited synteny ratios exceeding 95% (Figure 4B). For instance, chromosome 2 of *C. militaris* CH1 showed high synteny with chromosome 1 of *C. militaris* ATCC, chromosome 4 with chromosome 2, and chromosome 5 with chromosome 5. Additionally, chromosome fusion was observed between certain chromosomes of the two species. For example, the syntenic regions of chromosome 1 of *C. militaris* CH1 were distributed across chromosomes 1, 2, and 4 of *C. militaris* ATCC, while the syntenic regions of chromosome 3 of *C. militaris* CH1 were found on chromosomes 3 and 6 of *C. militaris* ATCC. In addition, chromosome inversion events were observed, such as the inversion between chromosome 7 of *C. militaris* CH1 and chromosome 7 of *C. militaris* ATCC.

Cluster analysis of the genes of *C. militaris* CH1 and *C. militaris* ATCC revealed a total of 8140 shared genes, with 908 and 90 genes unique to *C. militaris* CH1 and *C. militaris* ATCC, respectively (Figure 4C). To further compare the genomic similarity among different *Cordyceps* species, we also performed a comparison of *C. militaris* CH1, *C. militaris* ATCC, GCA_003332165.1, and GCF_000225605.1, where *C. militaris* CH1, GCA_003332165.1, and GCF_000225605.1 represent Chinese strains (Figure 4D). The results showed that the genomic similarity between all four strains was high, with all similarities exceeding 97%. Compared to *C. militaris* ATCC, *C. militaris* CH1 exhibited higher genomic similarity with both GCA_003332165.1 and GCF_000225605.1. Among them, GCF_000225605.1 showed the highest similarity to *C. militaris* CH1, with a similarity of 98.64%, while the similarity between *C. militaris* CH1 and *C. militaris* ATCC was 98.03% (Figure 4D). Further GO and KEGG enrichment analyses were performed on the genes unique to *C. militaris* CH1. The results revealed that these genes were significantly enriched in the GO terms of “negative regulation of biological process”, “reproductive process”, and “localization” (Figure 4E) as well as in the KEGG pathways “galactose metabolism”, “glycosphingolipid biosynthesis”, and “terpenoid backbone biosynthesis” (Figure 4F).

### 3.5. The Secondary Metabolism in C. militaris CH1

*C. militaris* CH1 possesses a large number of carbohydrate-active enzymes (CAZymes), with a total of 326 CAZymes identified in the *C. militaris* CH1 genome, which is 13 more than those found in *C. militaris* ATCC. This increase is mainly reflected in the numbers of glycoside hydrolases (GHs) and glycosyltransferases (GTs), which may contribute to the differences in quality between *C. militaris* CH1 and *C. militaris* ATCC. Additionally (Figure S4), 43 secondary metabolite gene clusters were identified in *C. militaris* CH1, including 7 T1PKS, 4 terpenes, 9 fungal-RiPPs, 15 NRPS, and 8 other metabolic clusters. Furthermore, a gene cluster related to cordycepin biosynthesis was identified on chromosome 3, consisting of four genes (*FUN_003262*, *FUN_003263*, *FUN_003264*, *FUN_003266*) involved in cordycepin synthesis (Figure S5). Four metabolic clusters were also found on chromosome 3, which may indirectly participate in cordycepin biosynthesis (Figure S6).

CYP450 enzymes play a critical role in secondary metabolism in fungi. To investigate the characteristics of the P450 family within the *C. militaris* genome, we identified 61 and 58 P450 genes in *C. militaris* CH1 and *C. militaris* ATAC, respectively. Phylogenetic analysis showed that the two strains of *C. militaris* have the same types of P450 families, with only slight differences in their numbers (Figure S7).

### 3.6. The Transcriptome and Metabolome Revealed the Effects of Light and Dark Conditions on the Mycelia of C. militaris CH1

**Figure 5:**
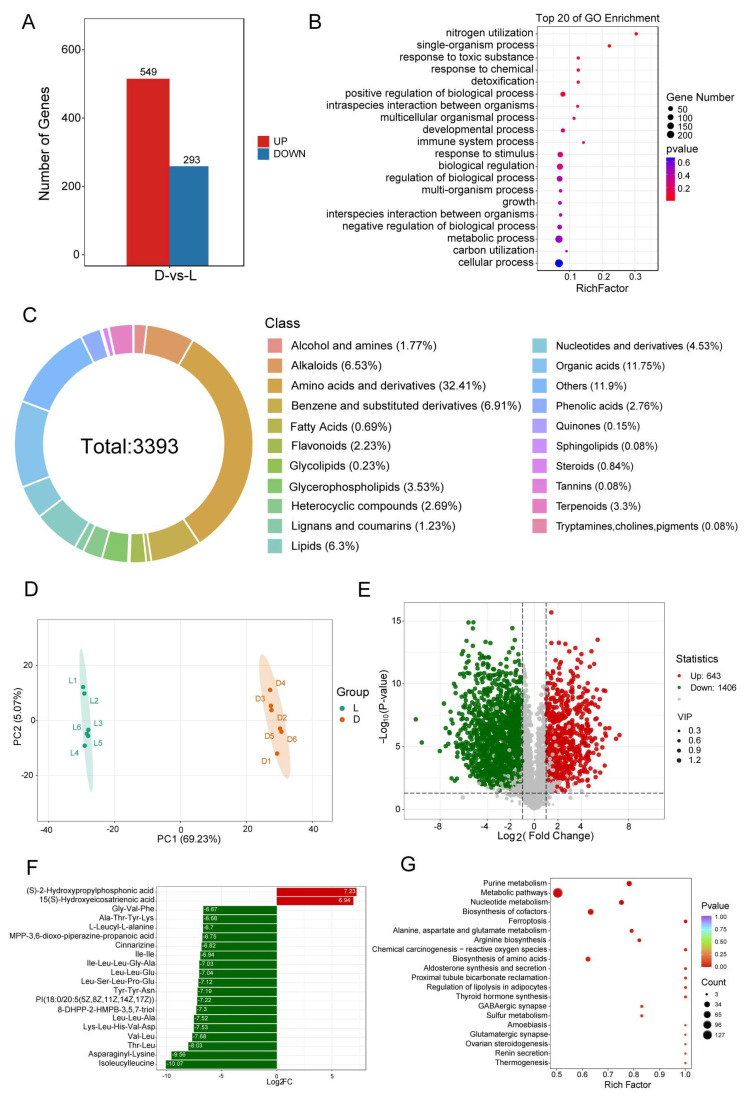
Transcriptome and metabolome analysis of mycelia growth of *C. militaris* CH1 under light and dark conditions. (**A**) The number of differentially expressed genes (upregulated and downregulated) under dark and light stress. (**B**) The top 20 GO enrichment items of differential genes. (**C**) Classification and proportion of all metabolites in *C. militaris* CH1 after light stress. (**D**) PCA analysis of control and treatment groups for metabolome analysis. (**E**) Volcanic maps of differential metabolites. (**F**) Ranked among the top 20 most significant variations in differential metabolites. (**G**) KEGG enrichment analysis of differential metabolites.

To investigate the gene expression patterns of *C. militaris* CH1 under light stress, we performed transcriptome sequencing on the mycelium after 5 days of light stress treatment, with mycelial growth under dark conditions as the reference. A total of 32.39 Gb of clean data were obtained (Table S12). Differentially expressed genes between the dark condition (control) and light stress (treatment) were identified, resulting in 842 differentially expressed genes, including 549 upregulated and 293 downregulated genes (Figure 5A). We found that four genes involved in cordycepin synthesis, as well as four adenine synthesis-related genes, exhibited significantly higher expression under light stress compared to dark stress (*FUN_003262*, *FUN_003263*, *FUN_003264*, *FUN_003266, FUN_000548*, *FUN_000593*, *FUN_005768*, *FUN_009691*). Moreover, to explore the functional roles of these differentially expressed genes, a functional enrichment analysis was conducted, revealing significant enrichment in GO items such as “nitrogen utilization”, “metabolic process”, “response to chemical”, and “positive regulation of biological process” (Figure 5B).

To investigate the metabolic changes in *C. militaris* CH1 under light stress, metabolite profiling was performed with dark conditions as the reference. Six biological replicates were selected for both the control and treatment groups, and PCA analysis showed significant metabolic differences between the samples, with clustering patterns consistent with their experimental grouping (Figure 5D). A total of 3993 metabolites were identified, with 2931 detected in positive ion mode and 1062 in negative ion mode (Figure 5C). The top five classes of metabolites by proportion were amino acids and derivatives, organic acids, benzene and substituted derivatives, alkaloids, and lipids, accounting for 32.41%, 11.75%, 6.91%, 6.53%, and 6.30%, respectively. Further analysis of differential metabolites revealed 2052 significantly altered metabolites, including 644 upregulated and 1408 downregulated metabolites (Figure 5E, Table S13). Among the top 20 most significantly changed metabolites, 18 were downregulated and 2 were upregulated (Figure 5F). Notably, 65% of these metabolites were amino acids and derivatives, including isoleucylleucine, asparaginyl-lysine, and threonyl-leucine, while the upregulated metabolites were organic acids (S)-2-hydroxypropylphosphonic acid and lipids 15(S)-hydroxyeicosatrienoic acid. KEGG enrichment analysis of these differential metabolites revealed significant enrichment in pathways such as “purine metabolism”, “metabolic pathways”, “nucleotide metabolism”, and “biosynthesis of cofactors” (Figure 5G).

### 3.7. The Combined Analysis of Transcriptomics and Metabolomics Elucidated the Cordycepin Biosynthesis Pathway in C. militaris CH1

**Figure 6:**
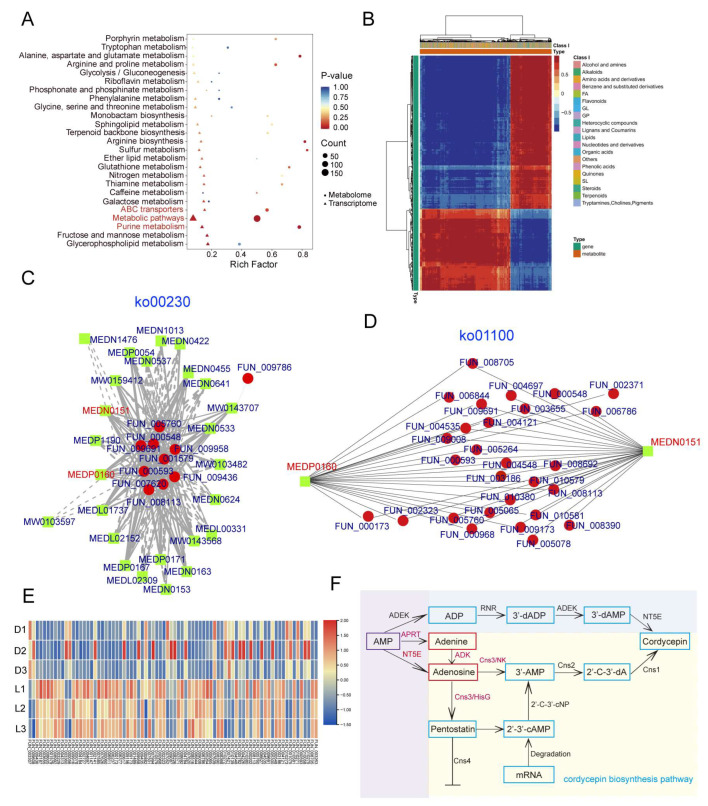
A combination of transcriptomic and metabolomic analysis of *C. militaris* CH1 under light stress. (**A**) KEGG pathways enriched by both genes and metabolites. (**B**) Heatmap showing the co-changes in genes and metabolites. (**C**) Interaction network of genes and metabolites in the purine synthesis pathway (ko00230). The red dots are target genes, the green dots are metabolites, and the connecting lines indicate the target genes required for the metabolites. (**D**) Interaction network of genes and metabolites involved in the synthesis of adenine and adenosine in the metabolic pathway (ko01100). (**E**) Heatmap of the expression levels of genes involved in the synthesis of adenine and adenosine. (**F**) Cordycepin biosynthesis pathway. Abbreviations: AMP: adenosine monophosphate; 3′-AMP: 3′-adenosine monophosphate; 2′,3′-cAMP: 2′,3′-cyclic adenosine monophosphate; ADK: adenosine kinase; ADEK, adenylate kinase; 3′-dAMP: 3′-deoxyadenosine monophosphate; ADP: adenosine diphosphate; 3′-dADP: 3′-deoxyadenosine diphosphates; 2′-C-3′-dA: 2 ‘-carbonyl -3′ -deoxyadenosine; RNR: ribonucleotide reductases; APRT, adenine phosphoribosyltransferase; NT5E, 5′-nucleotidase.

To investigate the relationship between genes and metabolites, a combined analysis of transcriptomic and metabolomic data from *C. militaris* CH1 under light stress was performed using the R packages clusterProfiler and pheatmap. The analysis was visualized in the form of dot plots, network diagrams, and heatmaps. The analysis revealed significant enrichment of several genes and metabolites in amino acid metabolism pathways (such as tryptophan, alanine, aspartate, glutamate, arginine, and proline) and secondary metabolite biosynthesis pathways (such as purine metabolism, terpenoid backbone biosynthesis, and porphyrin metabolism) in KEGG pathways (Figure 6A). Heatmap analysis of gene and metabolite expression patterns showed that both genes and metabolites responded collectively to light stress in *C. militaris* CH1, with some genes and metabolites showing similar expression trends, while others exhibited opposite trends (Figure 6B). Notably, we focused on analyzing the purine metabolism (ko00230) and metabolic pathways (ko01100) that may be involved in the synthesis of cordycepin (Figure 6C,D). Through correlation analysis, nine key enzyme genes involved in the synthesis of adenine and adenosine in the purine metabolism pathway were identified. Except for *FUN_009958*, whose expression was downregulated, the remaining eight enzyme genes showed an upregulation trend consistent with the changes in metabolite levels. In the metabolic pathway, 71 enzyme genes were identified as involved in the synthesis of adenine and adenosine. Among them, the expression of 46 genes was upregulated, consistent with changes in the levels of adenine and adenosine metabolites, while 26 genes showed downregulation (Figure 6E).

To identify genes and metabolites related to cordycepin biosynthesis, we conducted a comprehensive analysis of the transcriptomic and metabolomic data from *C. militaris* CH1 under light stress and identified differentially expressed genes (DEGs) and differentially abundant metabolites (DAMs) associated with the cordycepin biosynthesis pathway. We also constructed a metabolic pathway map for cordycepin biosynthesis (Figure 6F). The biosynthesis of cordycepin involves two main pathways: the biosynthesis of nucleosides/nucleotides and the biosynthesis of the single-gene clusters. Key enzymes involved in nucleoside/nucleotide biosynthesis include ribonucleotide reductase (RNR), 5′-nucleotidase (NT5E), and adenosine kinase (ADEK). The pathway primarily converts AMP to ADP through the action of ADEK, then generates 3′-deoxyadenosine-5′-diphosphate (3′-dADP) under the catalysis of RNR, followed by conversion to 3′-deoxyadenosine-5′-monophosphate (3′-dAMP) by ADEK. Finally, NT5E dephosphorylates 3′-dAMP to form cordycepin. We identified a set of single-gene clusters (*cns1–cns4*) in *C. militaris* CH1 associated with the cordycepin biosynthesis pathway, which includes *FUN_003266*, *FUN_003264*, *FUN_003263*, and *FUN_003262*. These genes encode a range of enzymes: oxidoreductase/dehydrogenase (cns1), metal-dependent phosphohydrolase (cns2), bifunctional nucleoside/nucleotide kinase and ATP phosphoribosyltransferase (cns3), and ABC multidrug transporter (cns4). Adenosine-3′-monophosphate (3′-AMP) is a direct precursor in the biosynthesis of cordycepin. One source of 3′-AMP is generated by the nucleoside/nucleotide kinase domain of Cns3 (Cns3-NK), which converts adenosine to 3′-AMP. The other source is the phosphorylation of 2′,3′-cyclic adenosine monophosphate (2′,3′-cAMP) catalyzed by 2′,3′-cyclic nucleotide phosphodiesterase (2′,3′-cNP). Subsequently, Cns2 catalyzes the conversion of 3′-AMP into the intermediate 2′-carbonyl-3′-deoxyadenosine (2′-C-3′-dA), which is then converted to cordycepin through an oxidoreductase reaction mediated by Cns1. Analysis of the expression changes in the four genes in *C. militaris* CH1 after light stress revealed a significant increase in *Cns3* expression, likely due to the increase in adenosine content.

### 3.8. qRT-PCR Analysis

**Figure 7:**
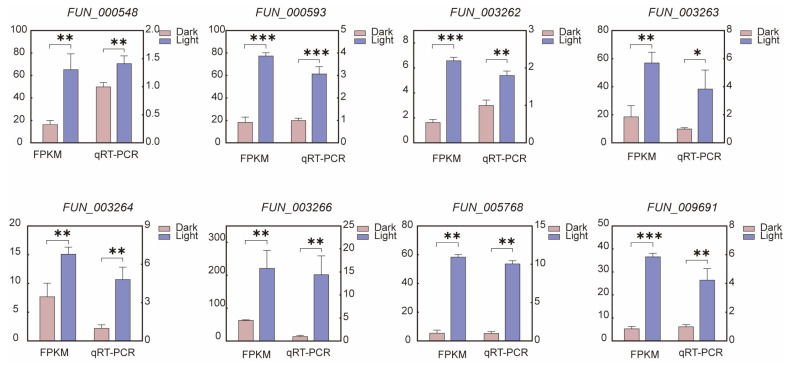
Verification of the cordycepin synthesis inferred from the transcriptome was conducted using qRT-PCR. Four genes related to cordycepin synthesis (*FUN_003262*, *FUN_003263*, *FUN_003264*, *FUN_003266*) and four genes involved in adenine synthesis (*FUN_000548*, *FUN_000593*, *FUN_005768*, *FUN_009691*) were selected for qRT-PCR analysis to validate the reliability of the RNA-seq results. The bar charts illustrate the FPKM values of these genes under light and dark conditions, as well as the relative expression levels calculated from qRT-PCR, with statistical significance between treatments denoted by asterisks: * indicates *p* < 0.05, ** indicates *p* < 0.01, and *** indicates *p* < 0.001.

To validate the accuracy of the transcriptomic data, we selected four genes related to cordycepin synthesis (*FUN_003262*, *FUN_003263*, *FUN_003264*, *FUN_003266*) and four adenine synthesis genes (*FUN_000548*, *FUN_000593*, *FUN_005768*, *FUN_009691*) for qRT-PCR analysis (Figure 7). The qRT-PCR results showed expression patterns that were in strong agreement with those obtained from RNA-seq, confirming the reliability and consistency of the transcriptomic analysis (Figure 7). The minor discrepancies in expression levels between the two methods can be attributed to differences in sensitivity and detection platforms.

## 4. Discussion

The complete sequencing of the T2T genome has opened new perspectives for fungal genomics, especially for fungal species with complex genome structures. Traditional genome sequencing methods often fail to effectively address the issues of repetitive sequences and telomeric regions, leading to incomplete genome assemblies that fail to capture the complexity of the genome. However, T2T genome sequencing, which combines high-precision long-read sequencing technologies (such as PacBio and Nanopore) with advanced assembly algorithms, successfully overcomes these technical challenges, greatly enhancing the completeness and accuracy of genome sequences. This method not only enables the acquisition of a complete fungal genome but also uncovers the intricate organization of repetitive and low-repetitive regions, providing essential insights into the evolution, functional distribution, and environmental adaptation of fungal genomes. For instance, T2T genome sequencing in *Trichoderma simmonsii* [60] and *Agrocybe chaxingu* [61] has revealed many important genomic regions related to environmental adaptation, drug resistance, and pathogenicity. These findings offer new perspectives on the survival mechanisms of fungi in natural environments and provide potential research directions for developing new agricultural biocontrol strategies and improving metabolic product synthesis.

In this study, we successfully constructed the T2T-level high-quality genome assembly of *Cordyceps militaris* CH1 by combining PacBio HiFi and Hi-C sequencing technologies. This approach enabled the successful assembly of all telomeres and centromeres across the seven chromosomes, and genome integrity assessments such as BUSCO demonstrated the high quality and accuracy of the genome assembly, providing a solid foundation for further research into the genetic characteristics and biological functions of *C. militaris* CH1. Compared to traditional genome assembly methods, T2T-level assembly effectively avoids assembly breaks and gaps caused by repetitive sequences and complex regions, offering a more accurate representation of the genome. Previous genome assemblies of *C. militaris* still had incomplete sequences or missing information in the chromosome ends and repetitive regions, which not only limited the comprehensive analysis of genome function but also affected the accuracy of evolutionary studies [14,15]. By using the T2T assembly method combining PacBio HiFi and Hi-C, this study successfully constructed a complete and seamless genome framework for *C. militaris* CH1, significantly improving genome quality compared to previous assemblies. This achievement provides a reliable basis for subsequent gene annotation, functional studies, and comparative genomics analysis and has significant implications for solving the challenges of long repetitive sequences and structural variations in fungal genomics research.

This study also conducted a comparative analysis of the genome of *C. militaris* CH1 with other *Cordyceps* species, particularly focusing on one strain from China and the other from the United States [16], to reveal their genomic differences and characteristics of system evolution. Firstly, through a comparison of the genomes of *C. militaris* CH1 (from China) and *C. militaris* ATCC (from the United States), although they belong to the same species, significant genetic differences were found, especially in gene family expansion and genomic structure. Single-nucleotide polymorphism (SNP) analysis showed a considerable number of SNPs between the two, with notable homologous region rearrangements observed on some chromosomes, such as chromosomal fusion and inversion events. In addition, the Cazymes and CYP450s identified in the *C. militaris* CH1 are slightly higher than *C. militaris* ATCC. These differences are likely related to geographical isolation, ecological adaptation, and the limitations of gene flow during species evolution. In the phylogenetic analysis, a gene family clustering analysis with 12 other fungal species showed that *C. militaris* CH1 shares a high degree of conservation in gene families with other *Cordyceps* species (such as *C. pruinosa* and *C. fumosorosea*), while some species-specific gene families were also identified. Based on the expansion of these gene families, we further inferred the evolutionary history of *C. militaris*, particularly that the expansion of gene families related to “stress response” and “secondary metabolism” pathways might be key to its adaptation to environmental pressures. The interaction between these gene families and environmental stimuli like light stress suggests a coordinated regulatory mechanism that allows *C. militaris* to rapidly adapt to changing conditions. Molecular clock analysis indicated that the divergence of *Cordyceps* species occurred approximately 43.12 Mya, while the divergence between *C. militaris* CH1 and ATCC strains occurred around 1.36 Mya, suggesting that *C. militaris* underwent significant evolutionary differentiation in a relatively short period. This finding provides a new perspective for understanding the evolutionary history of *Cordyceps* species, and further indicates that although *C. militaris* CH1 has relatively low genetic diversity due to its short evolutionary branch, it possesses considerable potential in adaptive evolution and the development of secondary metabolic pathways [62].

Through the combined analysis of transcriptomics and metabolomics, this study provides an in-depth exploration of the changes in genes and metabolites of *C. militaris* CH1 under light stress. The significant changes observed in the transcriptome and metabolome in response to light stress reflect the complex biological response of *C. militaris* CH1 to environmental pressures. The analysis revealed significant enrichment of genes and metabolites in amino acid metabolism and secondary metabolite biosynthesis pathways, particularly in KEGG pathways such as purine metabolism, terpenoid backbone biosynthesis, and chlorophyll metabolism, suggesting that these pathways may be closely related to the adaptive regulation of *C. militaris* CH1. The upregulation of genes involved in purine metabolism, like RNR and NT5E, indicates a shift in cellular energy dynamics, which is likely to contribute to the enhanced production of secondary metabolites like cordycepin under light stress. Previous studies have shown that light stress can significantly affect amino acid metabolism pathways in plants and fungi, especially the synthesis of amino acids such as tryptophan, glutamate, and arginine [63,64,65,66]. In this study, we observed that some genes and metabolites exhibited consistent expression patterns under light stress, indicating that they might respond to the stress within the same regulatory network. On the other hand, some genes and metabolites showed opposite trends, which may reflect the complex biological regulatory mechanisms, particularly the interactions between metabolic pathways. These findings suggest that the regulation of specific metabolic pathways under stress is probably part of a larger network that adjusts the cell’s response to external factors. Consistent with previous studies, the coordinated changes between genes and metabolites likely provide metabolic flexibility for *C. militaris* CH1 to cope with environmental stress, thereby ensuring the stability of its growth and product synthesis [67].

Cordycepin, one of the main active compounds in *C. militaris* CH1, is regulated by various factors, with light stress being one of the key regulators [68,69]. In this study, we focused on analyzing the metabolic pathways associated with cordycepin biosynthesis and found that light stress significantly regulates the expression of key enzyme genes in the purine metabolism and overall metabolic pathways, which in turn affects the synthesis of adenine and adenosine, thereby enhancing the efficiency of cordycepin synthesis. Specifically, the study showed that enzyme genes involved in adenine and adenosine synthesis (such as RNR, NT5E, and ADEK) were generally up-regulated under light stress, particularly in the purine metabolism pathway, which was consistent with the changes in the levels of adenine and adenosine metabolites. The increased expression of these genes may facilitate the accumulation of adenine precursors, accelerating the biosynthesis of cordycepin. The changes in the expression levels of these enzyme genes likely promote cordycepin biosynthesis by increasing the synthesis of adenosine precursors. Notably, adenosine-3′-monophosphate (3′-AMP), a direct precursor of cordycepin, is regulated by key genes such as *Cns3* and *Cns2*, whose upregulation under light stress may accelerate the accumulation of adenosine precursors, driving the final step of cordycepin synthesis. In summary, light stress influences the synthesis of adenine and adenosine by regulating key enzymes in multiple metabolic pathways, ultimately promoting the accumulation of cordycepin. This process is consistent with previous research [11]. These findings provide new insights into the regulation of light stress on the biosynthesis of *C. militaris* CH1 metabolites and offer potential regulatory strategies for the efficient production of cordycepin.

## 5. Conclusions

This study systematically explored the biological responses of *C. militaris* CH1 to light stress through genome sequencing, annotation, and integrated transcriptomic and metabolomic analyses. First, we successfully constructed a high-quality chromosomal-level genome and used gene family clustering analysis to reveal the evolutionary relationship between *C. militaris* CH1 and other *Cordyceps* species. In the light stress experiment, both the transcriptome and metabolome of *C. militaris* CH1 exhibited significant changes. Transcriptomic analysis identified 842 differentially expressed genes, while metabolomic analysis revealed 3993 metabolites, with 2052 showing significant changes. Combined transcriptomic and metabolomic analysis indicated that light stress regulates amino acid and purine metabolism pathways, particularly genes involved in adenine synthesis, promoting the biosynthesis of cordycepin. In summary, this study presents the complete T2T genome data of *C. militaris* CH1 and provides an in-depth understanding of the regulatory effects of light stress on gene expression and metabolite synthesis, offering a theoretical basis for studying and optimizing the biosynthesis pathways of cordycepin.

## Figures and Tables

**Table 1:** Analysis of the genetic architecture of the *C. militaris* CH genome.

Characteristics	Value
Total genes	10,749
Avg. gene length	1976.97
Avg. gene cds length	1540.18
Total cds	29,460
Total cds length	16,555,424
Avg. cds length	561.96
Avg. cds per gene	2.74
Total exons	30,942
Total exon length	22,116,766
Avg. exon length	714.78
Avg. exons per gene	2.88
Total genes with introns	7826
Total introns	19,367
Total intron length	1,538,583
Avg. intron length	79.44
Avg. introns per gene	1.8

## Data Availability

The *C. militaris* CH1 genome project had been submitted to GenBank SRA (PRJNA1215567). RNA-seq data have been submitted to GeneBank SRA (PRJNA1215690). The Cordyceps militaris CH1 genome assembly data were uploaded to the NCBI project PRJNA1251549.

## Supplementary Materials

The following supporting information can be downloaded at: https://www.mdpi.com/article/10.3390/jof11060461/s1, Figure S1. Depth map of the genome sequencing of *Cordyceps militaris* CH1 (divided using a 100k window); Figure S2. BUSCO scores of the genomes of four different species of *Cordyceps militaris* (*C. militaris* CH1, *C. militaris* ATCC, GCA_003332165.1, and GCF_000225605.1); Figure S3. Statistical distribution chart of SNP and INDEL positions for *C. militaris* CH1 and *C. militaris* ATCC; Figure S4. Statistical analysis of the number of carbohydrate-active enzymes (Cazymes) in *C. militaris* CH1 and *C. militaris* ATCC, along with a heatmap depicting the counts across different categories; Figure S5. Chromosomal distribution of gene clusters related to cordycepin synthesis; Figure S6. The secondary metabolite cluster of *Cordyceps militaris* on chromosome 3; Figure S7. Evolutionary tree of P450 enzymes in *Cordyceps militaris*; Table S1. QRT-PCR primers sequence; Table S2: The HiFi-reads of *C. militaris*; Table S3: The Hi-C data of *C. militaris*; Table S4: The final assembled genome of *C. militaris*; Table S5: The T2T assembled genome of *C. militaris*; Table S6: The BUSCO analysis with the Fungi_odb10 of T2T genome; Table S7: Statistics on Small RNAs in *C. militaris* genome; Table S8: Statistics on gene function annotation in *C. militaris* genome; Table S9: Classification of repetitive elements in the genome of *C. militaris*; Table S10: Cluster analysis of 12 fungi gene families based on genomic data. Table S11: Comparative analysis of different *C. militaris* genomes; Table S12: Transcriptome sequencing data of mycelium of *C. militaris* subjected to 5-day treatment under normal light and dark stress conditions; Table S13: The differential metabolite data of mycelium of *C. militaris* subjected to 5-day treatment under normal light and dark stress conditions.

## References

[1] Olatunji O.J., Tang J., Tola A., Auberon F., Oluwaniyi O., Ouyang Z. (2018). The genus *Cordyceps*: An extensive review of its traditional uses, phytochemistry and pharmacology. Fitoterapia.

[2] Shrestha B., Zhang W., Zhang Y., Liu X. (2012). The medicinal fungus *Cordyceps militaris*: Research and development. Mycol. Prog..

[3] Cui J.D. (2015). Biotechnological production and applications of *Cordyceps militaris*, a valued traditional Chinese medicine. Crit. Rev. Biotechnol..

[4] Das S.K., Masuda M., Sakurai A., Sakakibara M. (2010). Medicinal uses of the mushroom *Cordyceps militaris*: Current state and prospects. Fitoterapia.

[5] Zhang J., Wen C., Duan Y., Zhang H., Ma H. (2019). Advance in *Cordyceps militaris* (Linn) Link polysaccharides: Isolation, structure, and bioactivities: A review. Int. J. Biol. Macromol..

[6] Basith M., Madelin M. (1968). Studies on the production of perithecial stromata by *Cordyceps militaris* in artificial culture. Can. J. Bot..

[7] Dong C., Guo S., Wang W., Liu X. (2015). Cordyceps industry in China. Mycology.

[8] Jędrejko K.J., Lazur J., Muszyńska B. (2021). *Cordyceps militaris*: An overview of its chemical constituents in relation to biological activity. Foods.

[9] Cunningham K., Manson W., Spring F., Hutchinson S. (1950). Cordycepin, a metabolic product isolated from cultures of *Cordyceps militaris* (Linn.) Link. Nature.

[10] Xia Y., Luo F., Shang Y., Chen P., Lu Y., Wang C. (2017). Fungal cordycepin biosynthesis is coupled with the production of the safeguard molecule pentostatin. Cell Chem. Biol..

[11] Yang L., Li G., Chai Z., Gong Q., Guo J. (2020). Synthesis of cordycepin: Current scenario and future perspectives. Fungal Genet. Biol..

[12] Duan X., Yang H., Wang C., Liu H., Lu X., Tian Y. (2023). Microbial synthesis of cordycepin, current systems and future perspectives. Trends Food Sci. Technol..

[13] Jiaojiao Z., Fen W., Kuanbo L., Qing L., Ying Y., Caihong D. (2018). Heat and light stresses affect metabolite production in the fruit body of the medicinal mushroom *Cordyceps militaris*. Appl. Microbiol. Biotechnol..

[14] Zheng P., Xia Y., Xiao G., Xiong C., Hu X., Zhang S., Zheng H., Huang Y., Zhou Y., Wang S. (2012). Genome sequence of the insect pathogenic fungus *Cordyceps militaris*, a valued traditional Chinese medicine. Genome Biol..

[15] Chen Y., Wu Y., Liu L., Feng J., Zhang T., Qin S., Zhao X., Wang C., Li D., Han W. (2019). Study of the whole genome, methylome and transcriptome of *Cordyceps militaris*. Sci. Rep..

[16] Kramer G.J., Nodwell J.R. (2017). Chromosome level assembly and secondary metabolite potential of the parasitic fungus *Cordyceps militaris*. BMC Genom..

[17] Nurk S., Koren S., Rhie A., Rautiainen M., Bzikadze A.V., Mikheenko A., Vollger M.R., Altemose N., Uralsky L., Gershman A. (2022). The complete sequence of a human genome. Science.

[18] Naish M., Alonge M., Wlodzimierz P., Tock A.J., Abramson B.W., Schmücker A., Mandáková T., Jamge B., Lambing C., Kuo P. (2021). The genetic and epigenetic landscape of the Arabidopsis centromeres. Science.

[19] Wei C., Gao L., Xiao R., Wang Y., Chen B., Zou W., Li J., Mace E., Jordan D., Tao Y. (2024). Complete telomere-to-telomere assemblies of two sorghum genomes to guide biological discovery. iMeta.

[20] Song J.-M., Xie W.-Z., Wang S., Guo Y.-X., Koo D.-H., Kudrna D., Gong C., Huang Y., Feng J.-W., Zhang W. (2021). Two gap-free reference genomes and a global view of the centromere architecture in rice. Mol. Plant.

[21] Wang M., Meng G., Yang Y., Wang X., Xie R., Dong C. (2023). Telomere-to-Telomere Genome Assembly of Tibetan Medicinal Mushroom *Ganoderma leucocontextum* and the First *Copia* Centromeric Retrotransposon in Macro-Fungi Genome. J. Fungi.

[22] Wang Y., Yang L., Yang Q., Dong J., Wang Y., Duan Y., Yin W., Zheng L., Sun W., Fan J. (2022). Gap-free nuclear and mitochondrial genomes of Ustilaginoidea virens JS60-2, a fungal pathogen causing rice false smut. Mol. Plant-Microbe Interact..

[23] Huang X., Duan N., Xu H., Xie T., Xue Y.-R., Liu C.-H. (2018). CTAB-PEG DNA extraction from fungi with high contents of polysaccharides. Mol. Biol..

[24] Cheng H., Concepcion G.T., Feng X., Zhang H., Li H. (2021). Haplotype-resolved de novo assembly using phased assembly graphs with hifiasm. Nat. Methods.

[25] Hu J., Fan J., Sun Z., Liu S. (2020). NextPolish: A fast and efficient genome polishing tool for long-read assembly. Bioinformatics.

[26] Zeng X., Yi Z., Zhang X., Du Y., Li Y., Zhou Z., Chen S., Zhao H., Yang S., Wang Y. (2024). Chromosome-level scaffolding of haplotype-resolved assemblies using Hi-C data without reference genomes. Nat. Plants.

[27] Durand N.C., Robinson J.T., Shamim M.S., Machol I., Mesirov J.P., Lander E.S., Aiden E.L. (2016). Juicebox provides a visualization system for Hi-C contact maps with unlimited zoom. Cell Syst..

[28] Manni M., Berkeley M.R., Seppey M., Simão F.A., Zdobnov E.M. (2021). BUSCO update: Novel and streamlined workflows along with broader and deeper phylogenetic coverage for scoring of eukaryotic, prokaryotic, and viral genomes. Mol. Biol. Evol..

[29] Marçais G., Kingsford C. (2011). A fast, lock-free approach for efficient parallel counting of occurrences of k-mers. Bioinformatics.

[30] Vurture G.W., Sedlazeck F.J., Nattestad M., Underwood C.J., Fang H., Gurtowski J., Schatz M.C. (2017). GenomeScope: Fast reference-free genome profiling from short reads. Bioinformatics.

[31] Flynn J.M., Hubley R., Goubert C., Rosen J., Clark A.G., Feschotte C., Smit A.F. (2020). RepeatModeler2 for automated genomic discovery of transposable element families. Proc. Natl. Acad. Sci. USA.

[32] Tarailo-Graovac M., Chen N. (2009). Using RepeatMasker to identify repetitive elements in genomic sequences. Curr. Protoc. Bioinform..

[33] Stanke M., Keller O., Gunduz I., Hayes A., Waack S., Morgenstern B. (2006). AUGUSTUS: Ab initio prediction of alternative transcripts. Nucleic Acids Res..

[34] Korf I. (2004). Gene finding in novel genomes. BMC Bioinform..

[35] Keilwagen J., Hartung F., Grau J. (2019). GeMoMa: Homology-based gene prediction utilizing intron position conservation and RNA-seq data. Gene Prediction: Methods and Protocols.

[36] Haas B.J., Salzberg S.L., Zhu W., Pertea M., Allen J.E., Orvis J., White O., Buell C.R., Wortman J.R. (2008). Automated eukaryotic gene structure annotation using EVidenceModeler and the Program to Assemble Spliced Alignments. Genome Biol..

[37] Ashburner M., Ball C.A., Blake J.A., Botstein D., Butler H., Cherry J.M., Davis A.P., Dolinski K., Dwight S.S., Eppig J.T. (2000). Gene ontology: Tool for the unification of biology. Nat. Genet..

[38] Kanehisa M., Goto S. (2000). KEGG: Kyoto encyclopedia of genes and genomes. Nucleic Acids Res..

[39] Nawrocki E.P., Eddy S.R. (2013). Infernal 1.1: 100-fold faster RNA homology searches. Bioinformatics.

[40] Chan P.P., Lin B.Y., Mak A.J., Lowe T.M. (2021). tRNAscan-SE 2.0: Improved detection and functional classification of transfer RNA genes. Nucleic Acids Res..

[41] Madden T. (2013). The BLAST sequence analysis tool. NCBI Handb..

[42] Emms D.M., Kelly S. (2019). OrthoFinder: Phylogenetic orthology inference for comparative genomics. Genome Biol..

[43] Katoh K., Standley D.M. (2013). MAFFT multiple sequence alignment software version 7: Improvements in performance and usability. Mol. Biol. Evol..

[44] Minh B.Q., Schmidt H.A., Chernomor O., Schrempf D., Woodhams M.D., Von Haeseler A., Lanfear R. (2020). IQ-TREE 2: New models and efficient methods for phylogenetic inference in the genomic era. Mol. Biol. Evol..

[45] Yang Z. (2007). PAML 4: Phylogenetic analysis by maximum likelihood. Mol. Biol. Evol..

[46] Mendes F.K., Vanderpool D., Fulton B., Hahn M.W. (2020). CAFE 5 models variation in evolutionary rates among gene families. Bioinformatics.

[47] Yu G., Wang L.G., Han Y., He Q.Y. (2012). clusterProfiler: An R package for comparing biological themes among gene clusters. Omics J. Integr. Biol..

[48] Marçais G., Delcher A.L., Phillippy A.M., Coston R., Salzberg S.L., Zimin A. (2018). MUMmer4: A fast and versatile genome alignment system. PLoS Comput. Biol..

[49] Wang Y., Tang H., DeBarry J.D., Tan X., Li J., Wang X., Lee T.-h., Jin H., Marler B., Guo H. (2012). MCScanX: A toolkit for detection and evolutionary analysis of gene synteny and collinearity. Nucleic Acids Res..

[50] Finn R.D., Clements J., Eddy S.R. (2011). HMMER web server: Interactive sequence similarity searching. Nucleic Acids Res..

[51] Cortés-Maldonado L., Marcial-Quino J., Gómez-Manzo S., Fierro F., Tomasini A. (2020). A method for the extraction of high quality fungal RNA suitable for RNA-seq. J. Microbiol. Methods.

[52] Chen S. (2023). Ultrafast one-pass FASTQ data preprocessing, quality control, and deduplication using fastp. iMeta.

[53] Kim D., Paggi J.M., Park C., Bennett C., Salzberg S.L. (2019). Graph-based genome alignment and genotyping with HISAT2 and HISAT-genotype. Nat. Biotechnol..

[54] Shumate A., Wong B., Pertea G., Pertea M. (2022). Improved transcriptome assembly using a hybrid of long and short reads with StringTie. PLoS Comput. Biol..

[55] Ghosh S., Chan C.-K.K. (2016). Analysis of RNA-Seq data using TopHat and Cufflinks. Plant Bioinformatics: Methods and Protocols.

[56] Li G., Jian T., Liu X., Lv Q., Zhang G., Ling J. (2022). Application of metabolomics in fungal research. Molecules.

[57] Smedsgaard J., Nielsen J. (2005). Metabolite profiling of fungi and yeast: From phenotype to metabolome by MS and informatics. J. Exp. Bot..

[58] Singh V.K., Mangalam A.K., Dwivedi S., Naik S. (1998). Primer premier: Program for design of degenerate primers from a protein sequence. Biotechniques.

[59] Pfaffl M.W. (2001). A new mathematical model for relative quantification in real-time RT-PCR. Nucleic Acids Res..

[60] Chung D., Kwon Y.M., Yang Y. (2021). Telomere-to-telomere genome assembly of asparaginase-producing *Trichoderma simmonsii*. BMC Genom..

[61] Chen X., Wei Y., Meng G., Wang M., Peng X., Dai J., Dong C., Huo G. (2024). Telomere-to-Telomere Haplotype-Resolved Genomes of Agrocybe chaxingu Reveals Unique Genetic Features and Developmental Insights. J. Fungi.

[62] Chai L., Li J., Guo L., Zhang S., Chen F., Zhu W., Li Y. (2024). Genomic and Transcriptome Analysis Reveals the Biosynthesis Network of Cordycepin in *Cordyceps militaris*. Genes.

[63] Dong J.Z., Lei C., Zheng X.J., Ai X.R., Wang Y., Wang Q. (2013). Light wavelengths regulate growth and active components of c ordyceps militaris fruit bodies. J. Food Biochem..

[64] Dong J., Liu M., Lei C., Zheng X., Wang Y. (2012). Effects of selenium and light wavelengths on liquid culture of *Cordyceps militaris* Link. Appl. Biochem. Biotechnol..

[65] Toldi D., Gyugos M., Darkó É., Szalai G., Gulyás Z., Gierczik K., Székely A., Boldizsár Á., Galiba G., Müller M. (2019). Light intensity and spectrum affect metabolism of glutathione and amino acids at transcriptional level. PLoS ONE.

[66] Oliveira I., Brenner E., Chiu J., Hsieh M.-H., Kouranov A., Lam H.-M., Shin M., Coruzzi G. (2001). Metabolite and light regulation of metabolism in plants: Lessons from the study of a single biochemical pathway. Braz. J. Med. Biol. Res..

[67] Yu Z., Fischer R. (2019). Light sensing and responses in fungi. Nat. Rev. Microbiol..

[68] Radhi M., Ashraf S., Lawrence S., Tranholm A.A., Wellham P.A.D., Hafeez A., Khamis A.S., Thomas R., McWilliams D., De Moor C.H. (2021). A systematic review of the biological effects of cordycepin. Molecules.

[69] Zhou X., Luo L., Dressel W., Shadier G., Krumbiegel D., Schmidtke P., Zepp F., Meyer C.U. (2008). Cordycepin is an immunoregulatory active ingredient of *Cordyceps sinensis*. Am. J. Chin. Med..

