# Supplementary figures and images for: Telomere-to-Telomere Assembly of the Cordyceps militaris CH1 Genome and Integrated Transcriptomic and Metabolomic Analyses Provide New Insights into Cordycepin Biosynthesis Under Light Stress
Source: J Fungi (Basel). 2025 Jun 18;11(6):461. doi: 10.3390/jof11060461 (PMC12194794; doi:10.3390/jof11060461)

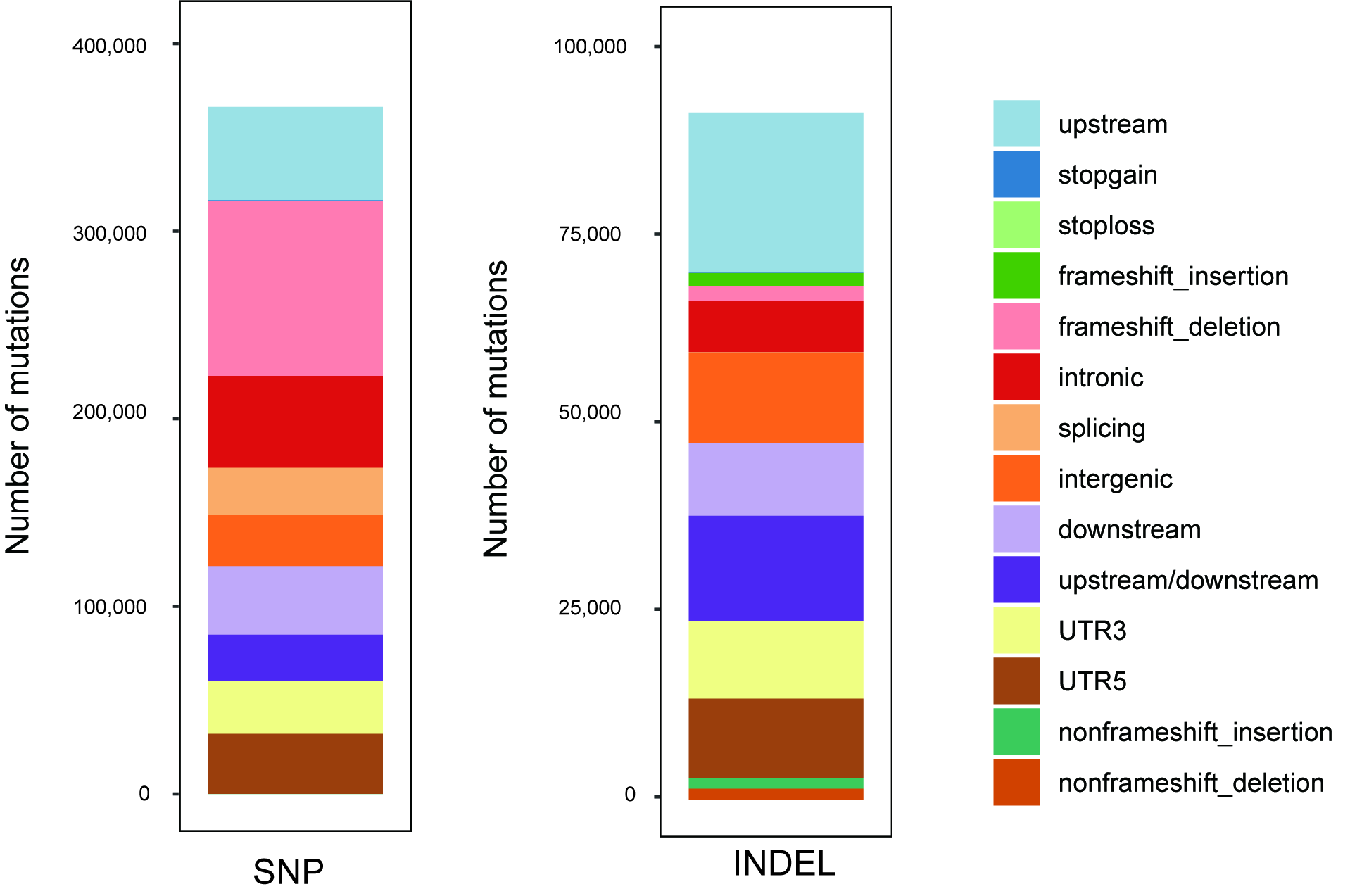

Supplement: Supplementary file 1 [file jof-11-00461-s001.zip › supplementary figure/S1.tif]

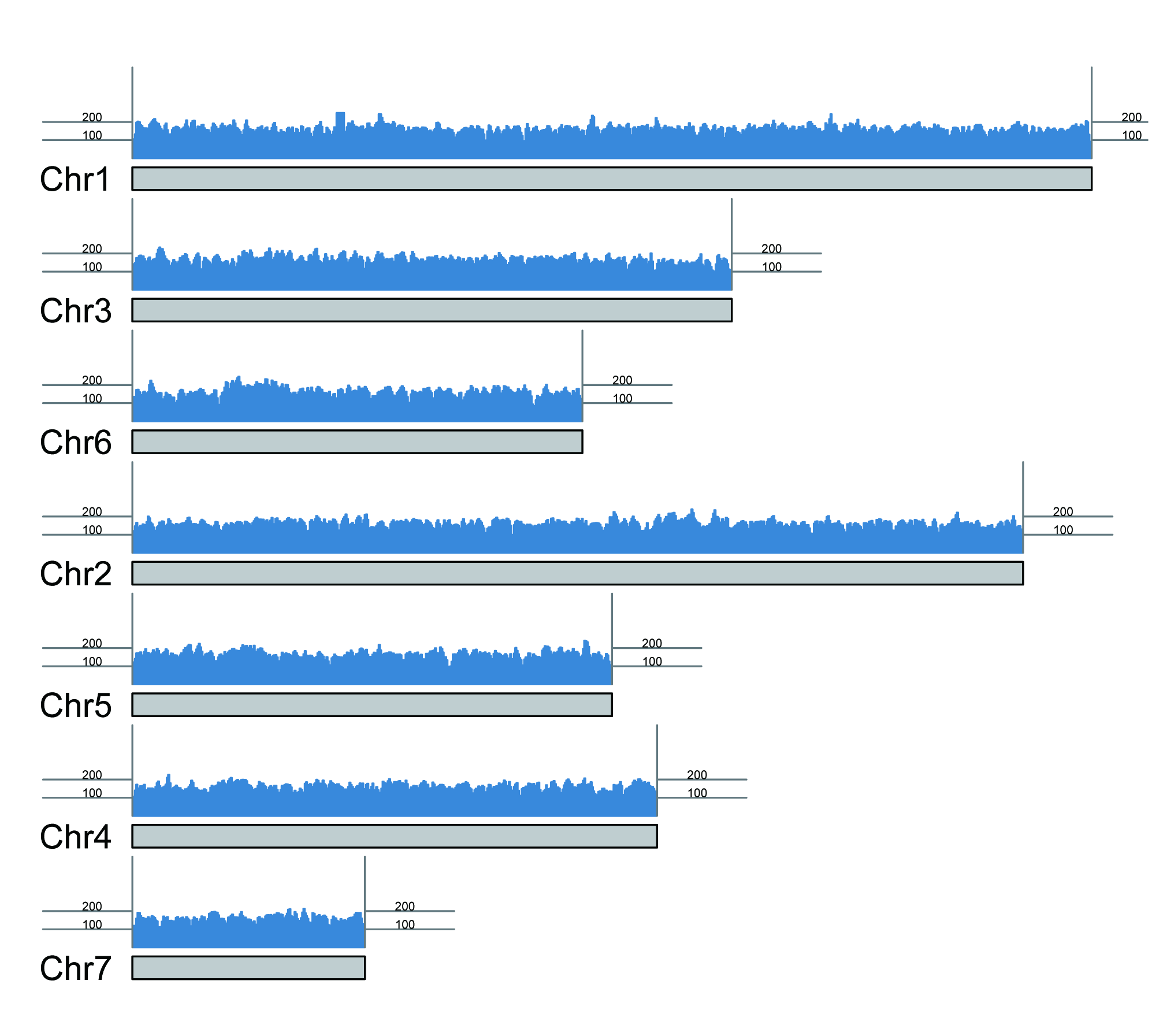

Supplement: Supplementary file 1 [file jof-11-00461-s001.zip › supplementary figure/S2.tif]

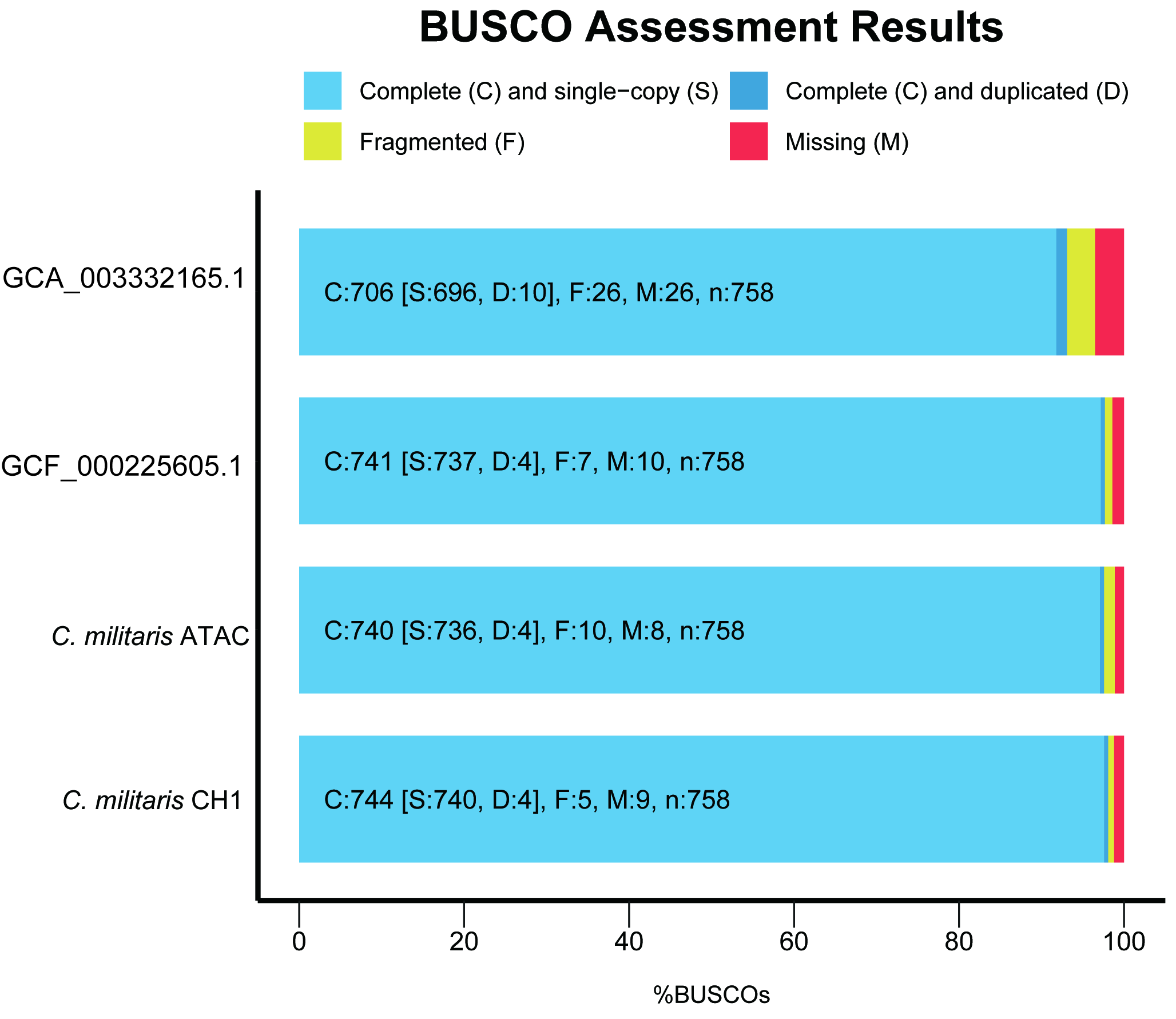

Supplement: Supplementary file 1 [file jof-11-00461-s001.zip › supplementary figure/S3.tif]

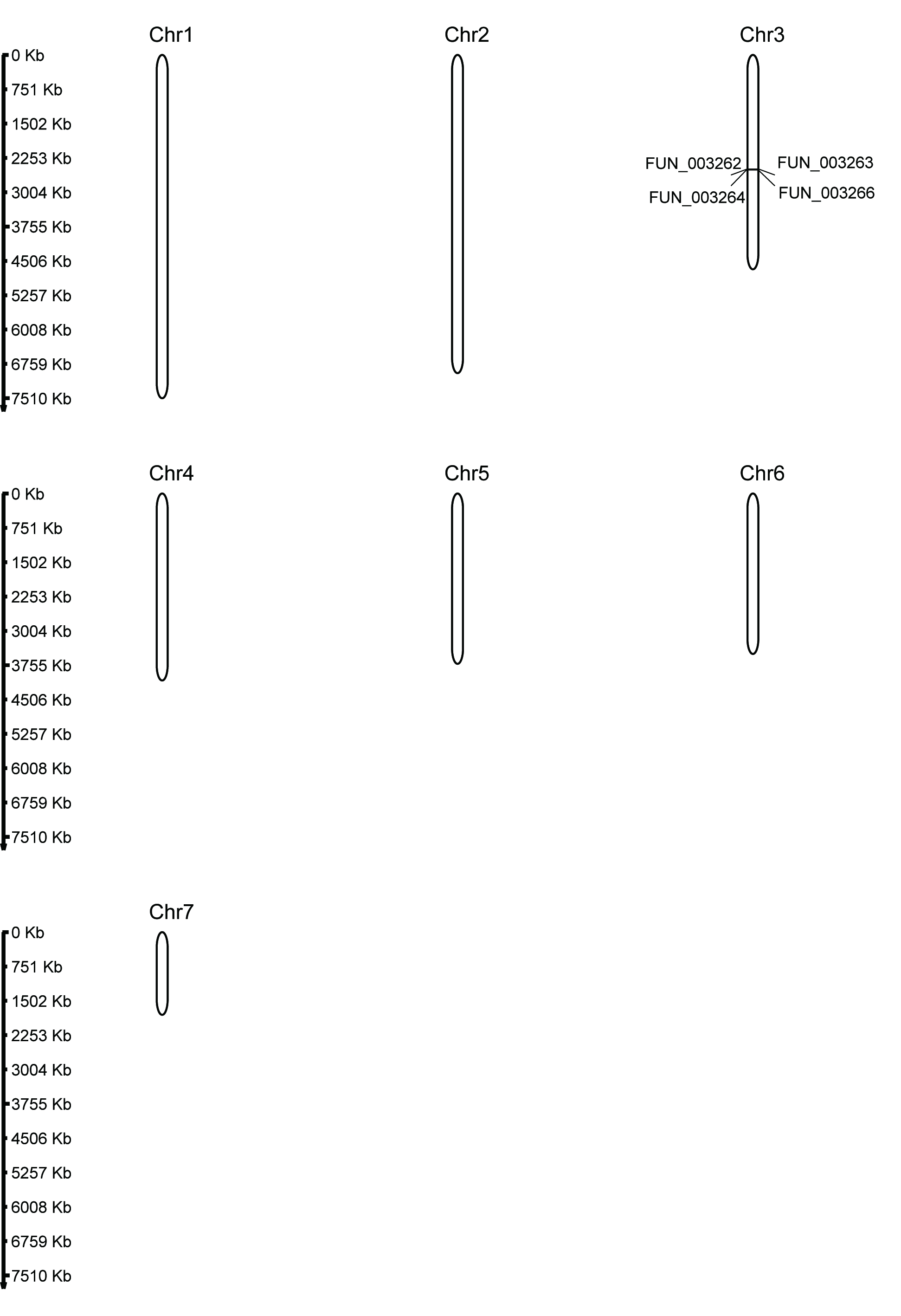

Supplement: Supplementary file 1 [file jof-11-00461-s001.zip › supplementary figure/S4.tif]

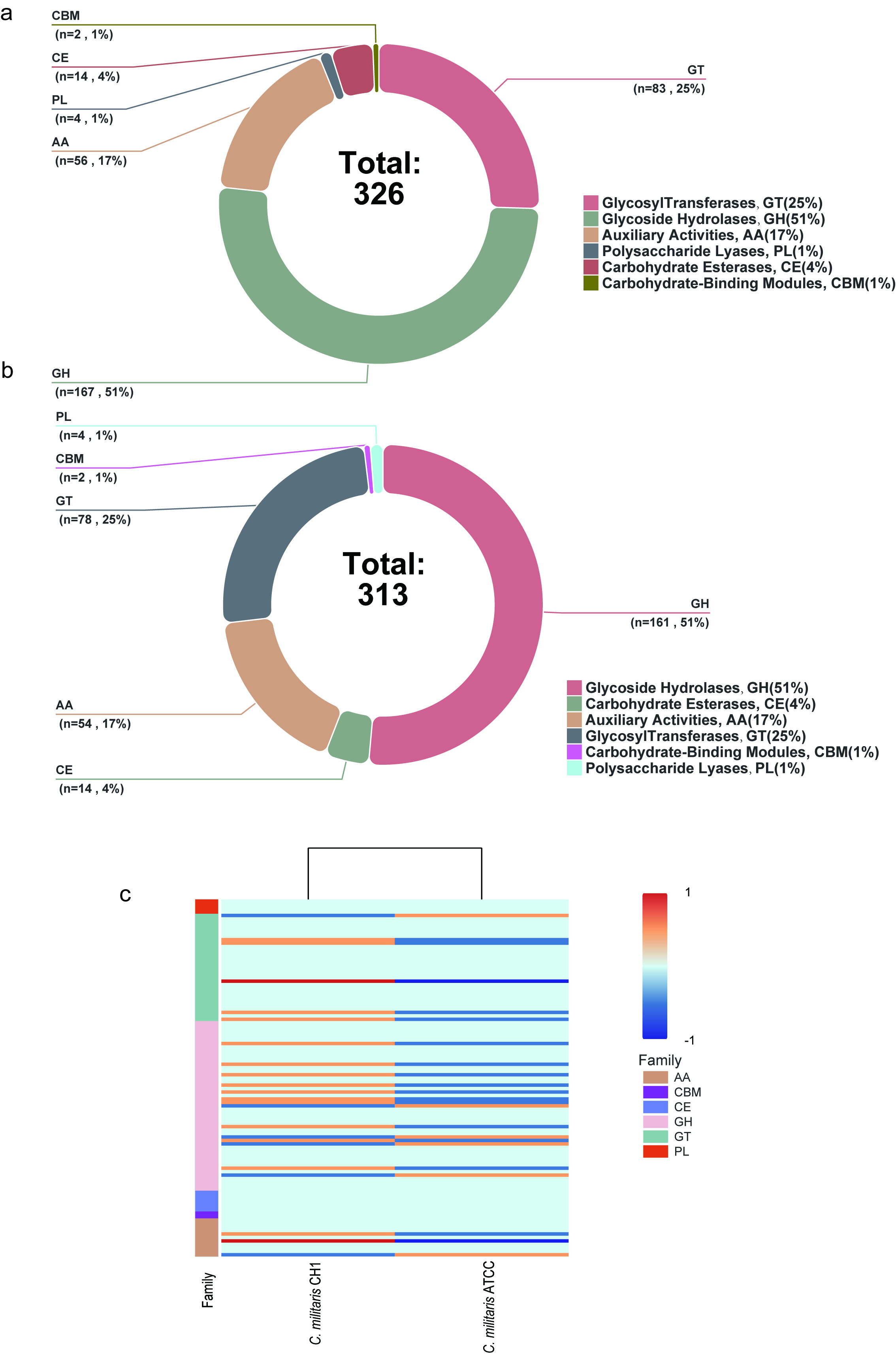

Supplement: Supplementary file 1 [file jof-11-00461-s001.zip › supplementary figure/S5-new.tif]

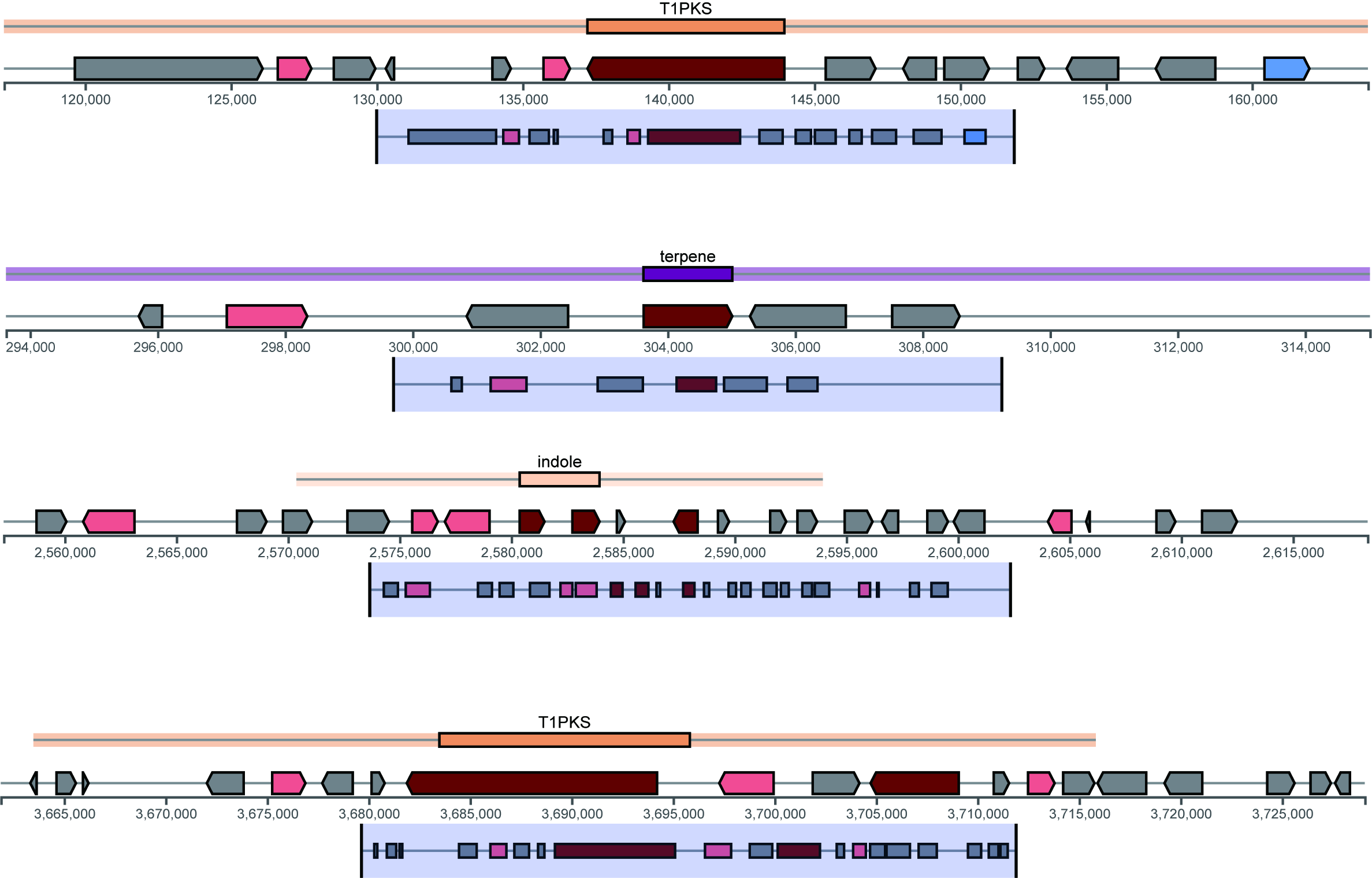

Supplement: Supplementary file 1 [file jof-11-00461-s001.zip › supplementary figure/S6.tif]

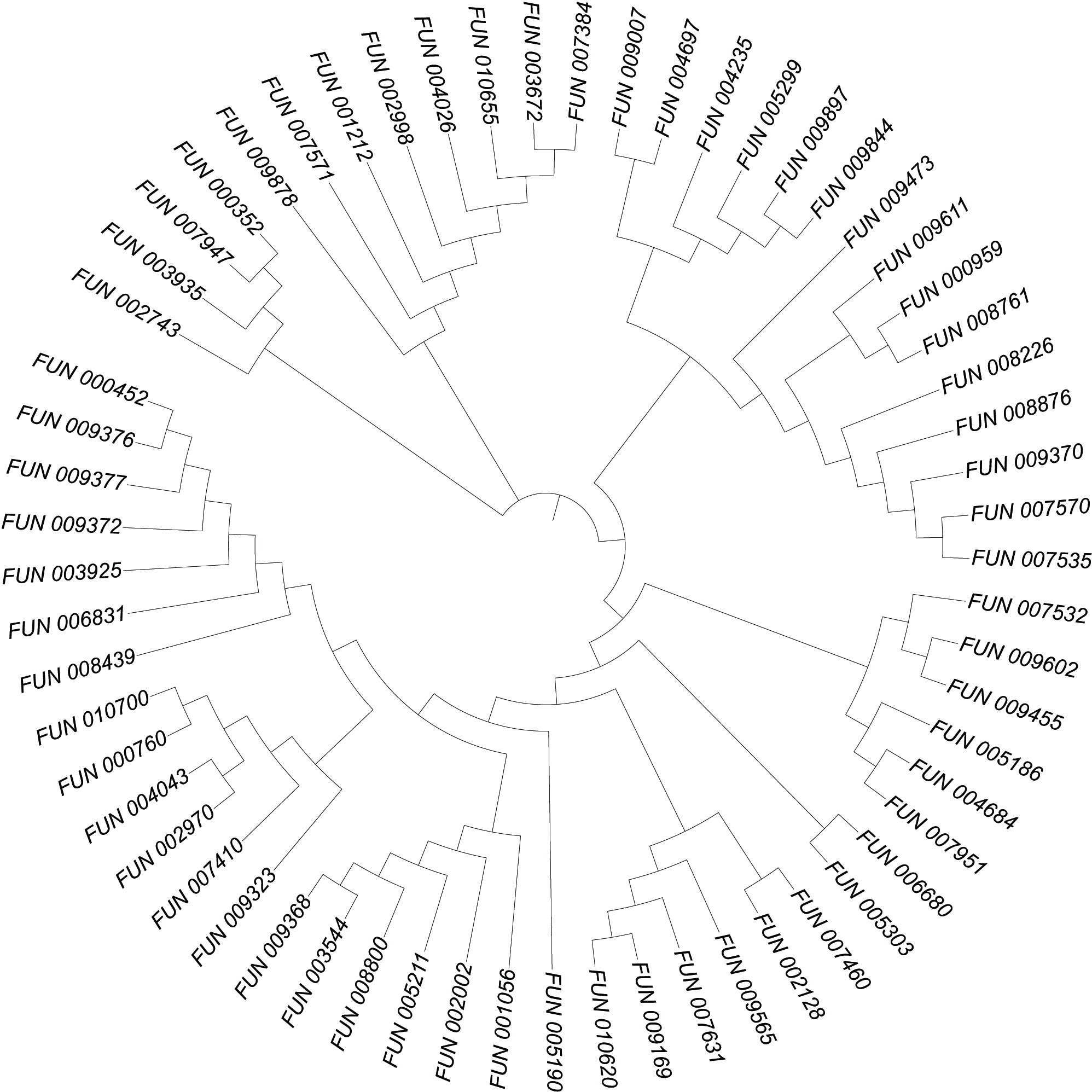

Supplement: Supplementary file 1 [file jof-11-00461-s001.zip › supplementary figure/S7.tif]
